# Modeling the spread of the Zika virus by sexual and mosquito transmission

**DOI:** 10.1371/journal.pone.0270127

**Published:** 2022-12-30

**Authors:** Saitel Agudelo, Mario Ventresca

**Affiliations:** 1 Universidad Nacional de Colombia, Bogota, Bogota, D.C, Colombia; 2 School of Industrial Engineering, Purdue University, West Lafayette, Indiana, United States of America; Texas A&M University College Station, UNITED STATES

## Abstract

Zika Virus (ZIKV) is a flavivirus that is transmitted predominantly by the *Aedes* species of mosquito, but also through sexual contact, blood transfusions, and congenitally from mother to child. Although approximately 80% of ZIKV infections are asymptomatic and typical symptoms are mild, multiple studies have demonstrated a causal link between ZIKV and severe diseases such as Microcephaly and Guillain Barré Syndrome. Two goals of this study are to improve ZIKV models by considering the spread dynamics of ZIKV as both a vector-borne and sexually transmitted disease, and also to approximate the degree of under-reporting. In order to accomplish these objectives, we propose a compartmental model that allows for the analysis of spread dynamics as both a vector-borne and sexually transmitted disease, and fit it to the ZIKV incidence reported to the National System of Public Health Surveillance in 27 municipalities of Colombia between January 1 2015 and December 31 2017. We demonstrate that our model can represent the infection patterns over this time period with high confidence. In addition, we argue that the degree of under-reporting is also well estimated. Using the model we assess potential viability of public health scenarios for mitigating disease spread and find that targeting the sexual pathway alone has negligible impact on overall spread, but if the proportion of risky sexual behavior increases then it may become important. Targeting mosquitoes remains the best approach of those considered. These results may be useful for public health organizations and governments to construct and implement suitable health policies and reduce the impact of the Zika outbreaks.

## 1 Introduction

The Zika Virus (ZIKV) is a flavivirus similar to dengue, chikungunya and yellow fever, that is primarily transmitted by *Aedes* mosquitoes [1]. It was first identified in Uganda in 1947 and isolated from human blood in 1952 [1, 2]. Large Zika outbreaks have been reported in Micronesia (2007), French Polynesia (2013–2014), Easter Island (2014), the Cook Islands (2014), New Caledonia (2014–2015), and Brazil (2015), spreading rapidly to other zones [2, 3]. Generally, the Zika Fever has mild symptoms including fever, rash, conjunctivitis, asthenia, myalgia, arthralgia and vomiting [4, 5], which can generally be treated at home and are present in a small proportion of infected individuals [6]. Approximately, 20% of those who have Zika reported symptoms [6, 7].

It was long assumed that ZIKV was a typical vector-borne disease, transmitted only by *Aedes* mosquitoes [8]. However, recent studies have reported and confirmed ZIKV transmission during pregnancy [9], through breastfeeding [10], blood transfusions [11], organ transplantation [12], sexual contact [13], and other rare nonsexual person-to-person mechanisms [14]. The virus has also been found in fluids such as urine [15, 16], saliva [16, 17], semen [16, 18], breast milk [19] and cervical mucus [20]. The reports on ZIKV horizontal transmission correspond to individuals who became infected through sexual contact with a person who acquired the virus in a country with autochthonous transmission of the virus [13], even if the symptoms are absent [21, 22]. Though most non-vector-borne ZIKV infections involve transmission from a man to his female partner [13], male-to-male [13, 23], and female-to-male [13, 24] transmission has also been reported.

While ZIKV can be transmitted by various pathways, the most influential with respect to overall disease spread are by mosquito and sexual contact. Concerning the latter, ZIKV has a causal relationship with Guillain Barré syndrome (GBS) [25], an uncommon disorder that damages the peripheral nervous system and can lead to paralysis [26]. Zika also increases the probability of occurrence of microcephaly, the condition of babies born with heads smaller than the average expected size and not fully developed brains [27]. The probability of acquiring some other birth defects also increases when the infection is acquired during pregnancy [25]. The distinctive set of neurological, brain, ocular, joint, muscle and cranial anomalies observed in newborns encompass the congenital Zika Syndrome (CZS) [28].

The research agenda of the World Health Organization (WHO) on prevention and control is focused on vaccine development, vector control, treatment and regulatory support [29]. Although great effort has been put in the development of a vaccine, it is not yet available [30]. However, given the complications associated with ZIKV and its rapid propagation, it is nonetheless imperative to reduce the risk of becoming infected. As a part of this endeavor, the WHO has published guidelines on sexual transmission prevention [31], vector control [32], antenatal care [33, 34], breastfeeding [35], blood transfusion safety [36], and aircraft disinfection [37]. Surveillance recommendations in regard to Zika, Guillain-Barré syndrome, microcephaly and other congenital anomalies were also published [38, 39]. Concerning laboratory testing, the WHO advocates for prioritization of symptomatic individuals, especially those with possible sexual contact acquired ZIKV infection [40]. Identical action should be taken towards pregnant women who might have been exposed to ZIKV and their newborns [40].

According to the WHO, as of February 2018, interrupted transmission with potential re-introduction of ZIKV has been observed in 15 areas in the Western Pacific Region and the Americas [41]. Ongoing transmission, on the other hand, has been reported in 71 countries and territories, including 12 areas in Africa, 42 in the Americas, 6 in South-East Asia, and 11 in the Western Pacific Region [41]. In Colombia, the Ministry of Health and Social Protection confirmed the autochthonous circulation of ZIKV in October of 2015 (epidemiological week (EW) 41, 2015), after finding the first nine cases in the department of Bolivar [42]. The end of the epidemic phase of the outbreak was declared in July of 2016 when a decrease in the number of ZIKV infections was observed [43]. Following the epidemiological alert emitted in December 2015, that warned about the Zika Virus and its correlation with neurological syndromes and congenital anomalies [44], the Colombian National Institute of Health (INS) requested all of the entities involved in the surveillance system to intensify the observation process of congenital defects and perinatal deaths due to congenital defects [45]. During the epidemic phase, 377.7 infections per 100,000 urban population individuals (UPI) were reported, and during the post-epidemic phase ZIKV incidence decreased to 18.2 infections per 100,000 UPI by the end of 2016 [46], and to 7.74 cases per 100,000 UPI by the end of 2017 [47]. The INS affirm that 18,117 women were pregnant during the epidemic phase of the outbreak [48]. Among the children born from those women, between January 2016 and the first week of May 2017, 316 cases of microcephaly and other congenital defects of the central nervous system associated to the Zika Virus have been confirmed [48]. In the endemic period, from the second week of May 2017 to the last week of June 2018, another 31 cases associated with the Zika Virus had been confirmed [48]. Colombia has also reported severe ZIKV infections leading to patient death [49, 50].

In the context of ZIKV circulation, various mathematical models and parameter estimations have been proposed [51–58]. The horizontal transmission of ZIKV was explored in [59] using a compartmental model that included human-to-human interactions under the assumption that asymptomatic individuals were not infectious, which has been disproved by [21, 22]. A subsequent approach [60] added asymptomatic individuals as contributors to the spread dynamics, but the model does not consider different periods of human-to-human and human-to-mosquito infectiousness, as [59]. Both of the aforementioned studies also assumed that symptomatic women and men had equal transmission probabilities and periods of infectiousness. The examination of scenarios where women-to-men and men-to-women transmission probabilities are different is relevant since more than 90% horizontal infections were reported as being male to female [13]. Moreover, ZIKV persistence in the genital fluids of male and female subjects has also been observed to be different [61, 62]. The model of [63] assumes equal women-to-men and men-to-women transmissibility, but incorporates different recovery rates for males and females. In a more recent study [64], all the considerations mentioned above are addressed, except for the fact that the model assumes that only men are able to infect other humans. None of the models considered the use of barrier methods in the population, which reduces the number of risky sexual encounters.

In this paper, we construct a compartmental model to represent the spread dynamics of ZIKV including its two main transmission pathways: mosquito bites and sexual contact. The structure of our model allows the simultaneous estimation of cases disaggregated by the presence of symptoms, transmission route, and sex. Symptomatic and asymptomatic individuals are able to spread the virus infecting mosquitoes and other humans. Following the kinetics of ZIKV, humans are infectious to mosquitoes for a shorter period than they are for other humans. Likewise, the infectious period of women and men is different and according to the persistence if the ZIKV is in semen or vaginal fluids. In consideration of the observed pattern of ZIKV sexual infections [13], women and men can infect each other with different transmission probabilities. Given that only one case of homosexual transmission has been documented [23], the model currently considers only heterosexual transmission.

In summary, the purpose of this study is multifaceted. One aim is to determine how significant is the contribution of the sexual transmission pathway to the spread dynamics of the ZIKV. To do this, we fit our model to the number of infections reported in 27 municipalities of Colombia [65–67] as reported over the three-year period between 2015 and 2017. Since the surveillance reports involve a degree of under-reporting, where the symptomatic cases who do not receive medical care and the asymptomatic infections are not likely detected by the surveillance services, we also aim to estimate the degree of under-reporting. We do this by including an expansion factor to estimate the symptomatic and overall incidence of the disease and the proportion of vector-borne and sexually acquired ZIKV infections. The third primary aim of this work is to examine the efficacy of vector control and sexual prevention health policies on mitigation of the disease spread, including the exploration of scenarios where the contribution of the sexual transmission pathway changes.

## 2 Materials and methods

### 2.1 Mathematical model

In order to assess the impact of the sexual transmission pathway we build upon the model presented in [68], by adding compartments and associated parameters to represent the virus persistence in the genital fluids and the interactions between women and men. The additional parameters increased model complexity and a two-stage procedure was utilized to avoid poor local optima when fitting. The model we propose, according to present knowledge of ZIKV [21, 22], allows sexual transmission between humans regardless of whether infectious individuals have symptoms or not. We do not consider mosquito recovery given that arbovirus infection of mosquitoes generally lasts for the vector lifespan [69].

**Fig 1:**
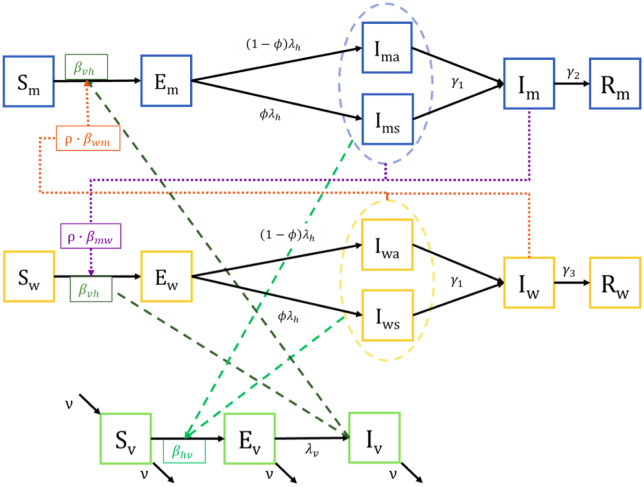
Mosquito and sexual transmission model for the Zika Virus. Susceptible men (*S*_*m*_) and susceptible women (*S*_*w*_) are exposed (*E*_*m*_, *E*_*w*_) to the virus. After the latent period, they become symptomatically infectious (*I*_*ms*_, *I*_*ws*_) or asymptomatically infectious (*I*_*ma*_, *I*_*wa*_). After 1/*γ*_1_ days the viral load is cleared from their blood, but can remain in their genital fluids (*I*_*m*_, *I*_*w*_), before recovering. Susceptible mosquitoes (*S*_*v*_) become exposed (*E*_*v*_) and, after the latent period, infectious (*I*_*v*_). Interactions between men, women and mosquitoes are depicted by broken lines.

Fig 1 illustrates the proposed compartmental model, which is based on a susceptible-exposed-infectious-recovered (SEIR) framework. Susceptible men (*S*_*m*_) are exposed (*E*_*m*_) to the virus through the bite of an infectious mosquito (*I*_*v*_) or sexual contact with infectious women (*I*_*wa*_, *I*_*ws*_, or *I*_*w*_). After the latent period (1/λ_*h*_), the men become infectious, and might (*I*_*ms*_) or might not (*I*_*ma*_) become symptomatic. The ZIKV disseminate in the blood and genital fluids of the infected individual. The mean time for clearance from blood is 1/*γ*_1_. The virus is cleared from semen and female fluids at a rate of 1/*γ*_2_ and 1/*γ*_3_ days, respectively, after clearance from blood. Therefore, men are able to infect mosquitoes and women for 1/*γ*_1_ days before the virus is cleared from their blood. Then, they remain infectious (*I*_*m*_) with viral load in semen, and are able to transmit the virus to women during 1/*γ*_2_ days, before they recover. Susceptible women (*S*_*w*_) are exposed (*E*_*w*_) to the virus through the bite of infectious mosquitoes (*I*_*v*_) or sexual contact with infectious men (*I*_*ma*_, *I*_*ms*_, or *I*_*m*_). After the latent period, the women become infectious, and might (*I*_*ws*_) or might not (*I*_*wa*_) develop symptoms. Women are able to infect mosquitoes and men for 1/*γ*_1_ days before the virus is cleared from their blood. Then, they remain infectious (*I*_*w*_) with viral load in vaginal secretions, and are able to transmit the virus to men during 1/*γ*_3_ days before they recover. A susceptible mosquito (*S*_*v*_) is exposed (*E*_*v*_) when it bites infectious humans who have a viral load in their blood (*I*_*ms*_, *I*_*ma*_, *I*_*ws*_, *I*_*wa*_). After the latent period, it becomes infectious (*I*_*v*_).

Eqs 1 to 16 describe ZIKV infectious dynamics as depicted in the mathematical model shown in Fig 1. Note that the cumulative number of symptomatic infections (*C*) is recorded when simulating the model and used to calculate the symptomatic incidence during model fitting.

$$\begin{array}{ccc} \frac{{\text{dS}}_{w}}{\text{dt}} & = & -\beta_{vh}S_{w}I_{v}-\beta_{mw}\rho S_{w}\left(I_{ma}+I_{ms}+I_{m}\right) \end{array}$$ (1)

$$\begin{array}{ccc} \frac{{\text{dE}}_{w}}{\text{dt}} & = & \beta_{vh}S_{w}I_{v}+\beta_{mw}\rho S_{w}\left(I_{ma}+I_{ms}+I_{m}\right)-\lambda_{h}E_{w} \end{array}$$ (2)

$$\begin{array}{ccc} \frac{{\text{dI}}_{\text{ws}}}{\text{dt}} & = & \phi \lambda_{h}E_{w}-\gamma_{1}I_{ws} \end{array}$$ (3)

$$\begin{array}{ccc} \frac{{\text{dI}}_{\text{wa}}}{\text{dt}} & = & \left(1-\phi \right)\lambda_{h}E_{w}-\gamma_{1}I_{wa} \end{array}$$ (4)

$$\begin{array}{ccc} \frac{{\text{dI}}_{w}}{\text{dt}} & = & \gamma_{1}\left(I_{ws}+I_{wa}\right)-\gamma_{3}I_{w} \end{array}$$ (5)

$$\begin{array}{ccc} \frac{{\text{dR}}_{w}}{\text{dt}} & = & \gamma_{3}I_{w} \end{array}$$ (6)

$$\begin{array}{ccc} \frac{{\text{dS}}_{m}}{\text{dt}} & = & -\beta_{vh}S_{m}I_{v}-\beta_{wm}\rho S_{m}\left(I_{wa}+I_{ws}+I_{w}\right) \end{array}$$ (7)

$$\begin{array}{ccc} \frac{{\text{dE}}_{m}}{\text{dt}} & = & \beta_{vh}S_{m}I_{v}+\beta_{wm}\rho S_{m}\left(I_{wa}+I_{ws}+I_{w}\right)-\lambda_{h}E_{m} \end{array}$$ (8)

$$\begin{array}{ccc} \frac{{\text{dI}}_{\text{ms}}}{\text{dt}} & = & \phi \lambda_{h}E_{m}-\gamma_{1}I_{ms} \end{array}$$ (9)

$$\begin{array}{ccc} \frac{{\text{dI}}_{\text{ma}}}{\text{dt}} & = & \left(1-\phi \right)\lambda_{h}E_{m}-\gamma_{1}I_{ma} \end{array}$$ (10)

$$\begin{array}{ccc} \frac{{\text{dI}}_{m}}{\text{dt}} & = & \gamma_{1}\left(I_{ms}+I_{ma}\right)-\gamma_{2}I_{m} \end{array}$$ (11)

$$\begin{array}{ccc} \frac{{\text{dR}}_{m}}{\text{dt}} & = & \gamma_{2}I_{m} \end{array}$$ (12)

$$\begin{array}{ccc} \frac{{\text{dS}}_{v}}{\text{dt}} & = & \nu N_{v}-\beta_{hv}S_{v}\left(I_{ms}+I_{ma}+I_{ws}+I_{wa}\right)-\nu S_{v} \end{array}$$ (13)

$$\begin{array}{ccc} \frac{{\text{dE}}_{v}}{\text{dt}} & = & \beta_{hv}S_{v}\left(I_{ms}+I_{ma}+I_{ws}+I_{wa}\right)-\lambda_{v}E_{v}-\nu E_{v} \end{array}$$ (14)

$$\begin{array}{ccc} \frac{{\text{dI}}_{v}}{\text{dt}} & = & \lambda_{v}E_{v}-\nu I_{v} \end{array}$$ (15)

$$\begin{array}{ccc} \frac{\text{dC}}{\text{dt}} & = & \lambda_{h}\phi \left(E_{m}+E_{w}\right) \end{array}$$ (16)

A summary of the parameter bounds are shown in Table 1. The rates of transmission between mosquitoes and humans is defined according to the convention of [70], which was also followed by [68] and discussed broadly with alternative approaches in [71]. Specifically, the transmission rate from humans to mosquitoes *β*_*hv*_ (Eq 17) is the product of the blood feeding rate (*a*), and the virus transmissibility to mosquitoes (*c*).

$$\begin{array}{c} \beta_{hv}=a\cdot c \end{array}$$ (17)

**Table 1:** Model parameters and their estimated realistic range.

Symbol	Definition	Range	Reference
*β*_*vh*_	Mosquito to human transmission rate	0.01–0.4	[59, 74, 77]
*β*_*hv*_	Human to mosquito transmission rate	0.09–0.5	[59]
*β*_*wm*_	Women to men transmission rate	0.001–0.1	[59]
*β*_*mw*_	Men to women transmission rate	0.01–0.1	[59]
*ϕ*	Proportion of symptomatic infections	0.10–0.27	[7]
1/λ_*h*_	Intrinsic latent period (days)	3–14	[81]
1/λ_*v*_	Extrinsic latent period (days)	8–12	[83]
1/*γ*_1_	Mean time to viral clearance from blood (days)	10–20	[88, 89]
1/*γ*_2_	Mean time to viral clearance from semen (days)	10–30	[89]
1/*γ*_3_	Mean time to viral clearance from vaginal fluids (days)	1–20	[61]
1/*ν*	Mosquito life span (days)	4–50	[83]

The transmission rate from mosquitoes to humans *β*_*vh*_ (Eq 18) is the product of the blood feeding rate (*a*), the virus transmissibility to humans (*b*), and the ratio of mosquitoes to humans (*m*).

$$\begin{array}{c} \beta_{vh}=m\cdot a\cdot b \end{array}$$ (18)

The ranges for *a*, *b* and *c* correspond to the values used in [59]. For *m*, the estimates of the ratio of female pupae per person were used, which is highly correlated to, and can be used to estimate, the risk of transmission [72, 73]. This ratio has also been reported in various survey studies conducted in different regions in Colombia [74–77].

The person-to-person transmission rates (*β*_*wm*_, *β*_*mw*_) are in the range suggested in [59] and is consistent with the reported transmission probability per coital act reported for HIV [78–80]. The model incorporates the assumption made in [55], that human latent period is equivalent to the intrinsic incubation period. The latent period of Zika (1/λ_*h*_), as considered in our model, is thus the number of days before an infected person becomes symptomatically or asymptomatically infectious. The intrinsic incubation period for the Zika Virus was estimated in [81, 82], using those confidence we assume a constant λ_*h*_ = 6.8.

Similarly, the latent period for mosquitoes (1/λ_*v*_) is the number of days before an infected mosquito becomes infectious. This interval was estimated in [83] for dengue, another *Aedes*-transmitted virus and is commonly used for ZIKV. In the model of [84] the similar confidence ranges are used. We follow this tradition, but assume a constant λ_*v*_ = 10, and also assume a constant mosquito life span of *ν* = 14 days.

According to the proposed mathematical model, an exposed individual becomes infectious with Zika viral load in blood and genital fluids. The viral clearance from blood occurs in (1/*γ*_1_). Whereas, the virus remains in semen for (1/*γ*_2_) days, and in vaginal fluids for (1/*γ*_3_) days. Most of the sexually transmitted ZIKV infections reported occurred between an infectious man and his partner [21, 22, 85–87]. Though female-to-male transmission has been reported [24], it is understudied. The Zika Virus persistence in blood (1/*γ*_1_) has been estimated to be between 10–20 days [88, 89], noting that the persistence of the ZIKV in semen has been examined in different studies [90–93] as well. The largest period of time that ZIKV has been detected in semen is six months after symptom onset [62]. The ZIKV persistence in semen estimates of a prospective cohort study in Puerto Rico [89] are often cited at 90 days, but given recent findings showing the efficacy of genital secretions from Zika infected men being extremely rare beyond 30 days [94], and in order to eliminate the contribution from outliers we use the median estimate reported (34 days (95%*CI*, 28 to 41). That is equivalent to a maximum of 20 days after viral clearance from blood (1/*γ*_2_). ZIKV has been detected in female genital secretions up to 37 days after onset of symptoms [61], but this has been rare to detect. As the persistence of ZIKV in the female track is still understudied, 20 days was used as the maximum duration of viral shedding in the genital secretions of symptomatic and asymptomatic women.

Although the Zika virus can be transmitted to people of all ages, only sexually active people can be horizontally infected. According to the Colombia Demographic and Health Survey 2015 [95–97] 56.1% of women aged 13–49 and 62.2% of men in the same age group are sexually active. Traditional contraception methods such as periodic abstinence, withdrawal, and lactational amenorrhea method (LAM), and modern contraception methods such as sterilization, oral hormonal pills, intra-uterine device (IUD), injectables, and implants are not effective protection against sexually transmitted infections, thus individuals using those methods are at risk of being sexually infected with the ZIKV. The aforementioned study determined that 5.8% of married or in-union women and 16.4% of single but sexually active women use condoms, for a total of 22.2% [95–97]. A higher percentage of use was observed for males, where 8.7% of married or in-union men and 46.0% of single but sexually active men reported to use condoms, totaling 54.7% [95–97].

Thus, in Eq 19 the proportion of risky sexual interactions in the population (*ρ*) was calculated as the sum of the proportion of sexually active women of reproductive age who do not use condoms (*W*_*nc*_) and the proportion of sexually active men of reproductive age who do not use condoms (*M*_*nc*_), multiplied by the contact rate (*CR*), which was assumed to be monogamous and 2.5 times per week, assuming slight variations from the estimates in [98].

$$\begin{array}{c} \rho =(W_{nc}+M_{nc})\cdot CR \end{array}$$ (19)

Approximately 7% of sexual interactions per person per day are risky encounters, which might lead to the horizontal transmission of the ZIKV. This calculation was made using the population estimates from years 1985–2005 and projections for 2005–2020 disaggregated by sex, area and five-year age groups [99] and the statistics of the Colombia Ministry of Health and Social Protection and the Association for the welfare of the Colombian Family (Profamilia) [95–97]. Table 2 shows the estimated proportion of risky sexual encounters per municipality, calculated as in the Eq 19.

**Table 2:** Estimated at risk population by municipality.

Code	Municipality	Department	Population at risk	Risky sexual encounters *ρ*(%)	Elevation (m)
8001	Barranquilla	Atlantico	210234	7.24	18
8758	Soledad	Atlantico	128930	7.40	58
13001	Cartagena	Bolivar	245239	7.30	2
18001	Florencia	Caqueta	36864	7.27	243
23001	Monteria	Cordoba	182250	7.33	50
41001	Neiva	Huila	57461	7.40	442
41298	Garzon	Huila	21342	6.96	829
47001	Santa Marta	Magdalena	126642	7.18	2
50001	Villavicencio	Meta	73525	7.48	467
50006	Acacias	Meta	13243	7.26	499
54001	Cucuta	Norte de Santander	143381	7.29	321
54405	Los Patios	Norte de Santander	13452	7.37	412
54874	Villa del Rosario	Norte de Santander	17247	7.41	441
68001	Bucaramanga	Santander	60300	7.29	959
68081	Barrancabermeja	Santander	42763	7.19	75
68276	Floridablanca	Santander	23708	7.39	926
68307	Giron	Santander	24142	7.37	706
68547	Piedecuesta	Santander	16858	7.32	1005
70001	Sincelejo	Sucre	107681	7.26	180
73001	Ibague	Tolima	76510	6.95	1286
76001	Cali	Valle del Cauca	243657	7.33	1019
76111	Guadalajara de Buga	Valle del Cauca	12237	6.96	969
76147	Cartago	Valle del Cauca	20170	6.77	918
76520	Palmira	Valle del Cauca	36748	7.22	1001
81001	Arauca	Arauca	27506	6.73	125
85001	Yopal	Casanare	30187	7.76	351
88001	San Andres	San Andres	28987	7	85

### 2.2 Data sources

The political and administrative division of Colombia includes departments, *intendencias*, *comisarías*, municipalities, and population centers. Considering the heterogeneity of the population and the availability of data, municipalities were selected as cases of study. In order to show the impact of sexual transmission in representative territories within the 1,122 municipalities in Colombia, the largest 27 municipalities by number of reported infections will be used. These 27 municipalities each had at least 500 reported infections and accounted for 69.65% of the total infections reported on a national level.

#### 2.2.1 Population at risk

The susceptible, exposed, infectious, and recovered compartments, are defined as proportions with respect to the population at risk (Nh), which is defined as the group of individuals residing in areas with average temperatures above 18°*C* and whose living conditions make them especially susceptible to vector-borne diseases. High temperatures are strongly correlated with a large number of vectors, and the occurrence of outbreaks [100]. Poverty, on the other hand, is associated with the lack of adequate infrastructure for the storage, distribution, and management of water and wastewater, which favors vector survival [101]. The number of individuals living in places with temperatures above 18°*C*, as calculated in [102], was scaled according to the Index of Unsatisfied Basic Needs published by the Colombian National Department of Statistics (DANE) [103]. This indicator is the result of the implementation of a methodology recommended by the United Nations Economic Commission for Latin America and the Caribbean (ECLAC) to identify severe deficiencies and characterize poverty in a population. An immunologically naive population with no prior immunity to ZIKV was assumed since this study encompasses the infections originated since the introduction of the ZIKV in Colombia in 2015 [42]. The model does not consider the impact of cross immunity; however, we consider it as a possible source of uncertainty in the discussion section.

The population of mosquitoes at risk was calculated using the proportions of female mosquitoes to humans reported in [73]. The proportion of women and men was calculated according to the population estimates from years 1985–2005 and projections for 2005–2020 disaggregated by sex, area, and five-year age groups, published by DANE [99].

Table 2 shows the code assigned to each municipality within the codification of the Political-Administrative Division (DIVIPOLA), the name of the municipality, the name of the department and the estimated corresponding human at-risk population.

#### 2.2.2 Weekly incidence

The used weekly incidence data was reported to the Colombian National Public Health Surveillance System (SIVIGILA) from 2015 to 2017 as General Zika Disease (code 895), which includes neurological disorders probably associated with a ZIKV infection, and is available to the public in [65–67]. Other ZIKV infections were reported as a part of broader epidemiological events: pregnant women were reported as Extreme Maternal Morbidity (code 549), newborns were reported as Congenital Defects (code 215) and the stillbirths and newborn deaths were reported as Perinatal or Late Neonatal Mortality (code 560) [104]. Although this data is also available to the public [65–67], it was not considered here because it is not presented with a specific cause, making it impossible to identify specific ZIKV cases.

### 2.3 Fitting procedure

A multi-step fitting strategy is used whereby the first step performs a global search to obtain a suitable set of parameters that then is used as the initial estimate for the second (local search) step of the fitting process. In the former step, the cumulative symptomatic incidence (*C*) is fit to the cumulative number of reported infections per week. The result is a set of parameters that do not describe the incidence of the ZIKV. For that reason, in the latter step the symptomatic incidence per week is fit to the number of reported infections per week. A quasi-Newton method [105] was used to find the best set of parameters in this second step. This optimization method runs port routines [106] that discover a vector **x** that minimizes a function *f(x)*, where **x** might be unconstrained or box-constrained. A bounds-constrained vector of parameters (see Table 1) is used to minimize a cost function, with constant values of λ_*h*_, λ_*v*_, *ν* and *γ*_1_ as indicated in the previous section. The cost function is calculated as the sum of squared weighted residuals as depicted in Eq 20, where *w* is the weight and a residual is the difference between the model prediction (*Mod*) and the number of cases reported (*Obs*).

$$\begin{array}{c} cost=\sum_{i=1}^{n}{\left(Mod_{i}-Obs_{i}\right)}^{2}\cdot w_{i}^{2} \end{array}$$ (20)

Because our model accounts for under-reporting, the weights are distributed so that the fitted points at the peak of the incidence curve have a greater penalty.

A Markov Chain Monte Carlo simulation that includes an adaptive Metropolis algorithm with a delayed rejection procedure is also leveraged to fit the data, which provides a confidence bound about its most probably parameterization. The method is executed for a maximum of 15000 iterations with a burn-in period of 3000 iterations.

The symptomatic incidence was fit to the reported infections, noting that the public health surveillance for the ZIKV in Colombia prior to and during the epidemic was based on the symptomatology exhibited by the patient [107, 108]. The confirmation of cases through laboratory tests was indicated for individuals coming from another country, individuals in areas without confirmed circulation of the virus and individuals in a risk group regardless of the area. The groups at risk include newborns (younger than two years of age), pregnant women, adults older than 65 years of age and individuals with co-morbidities. Clinical confirmation was indicated for individuals with Zika-like symptoms not attributable to other conditions who live in or come from areas with ongoing transmission of the virus.

The compartments of the model describe the prevalence of the ZIKV; not the incidence. In particular, the infectious compartments (*I*_*wa*_, *I*_*ws*_, *I*_*w*_, *I*_*ma*_, *I*_*ms*_, *I*_*m*_) describe the number of individuals that became infected at a given time (present or past) and remain infected at the current time. Taking that into account, the cumulative number of symptomatic infections per week (*C*) is recorded, and from that, it is possible to calculate the symptomatic incidence [55, 68].

#### 2.3.1 Under-reporting

In addition to estimating model parameters, the degree of under-reporting is also estimated. As discussed above, under-reporting seems likely to occur given that approximately 80% of ZIKV cases seem asymptomatic [7], and might pass unnoticed. Additionally, considering that when the symptoms are present they are usually mild [109], it is logical to presume that most infected individuals do not seek medical care. There are various caveats to using the approach outlined below, which will be highlighted in the Discussion Section. Nevertheless, expansion factors have provided useful insight when studying related diseases such as Dengue [110].

The general idea is to artificially boost observed data to levels one would observe with full reporting. In order to estimate the total number of symptomatic cases, the data from SIVIGILA [65–67] was modified by calculating an expansion factor (EF). First though, a multiplicative correction factor (CF) is applied to the reported cases prior to executing the fitting procedure. To determine this factor the fitting procedure was re-executed for multiple *CF*s, while estimating the best set of model parameters for each. The model parameters associated with the CF having minimum root mean square error (RMSE) was selected as the best fit. However, the CF alone does not properly reflect the under-reporting degree since it multiplies the number of symptomatic infections reported each week by the same quantity, with the best-fit parameters accounting for the remaining variation. An expansion factor for each municipality is therefore calculated as the ratio of estimated infections to reported symptomatic infections (*EF*_*s*_):

$$\begin{array}{c} EF_{s}=\frac{I_{estimated}}{I_{reported}} \end{array}$$ (21)

Then, an expansion factor (*EF*) for each of the 27 municipalities was calculated using the estimates for the proportion of symptomatic infections (*ϕ*) and the value of *EF*_*s*_:

$$\begin{array}{c} EF=\frac{EF_{s}}{\phi } \end{array}$$ (22)

Hence, if the model is perfectly fit to reported data then *CF* = *EF*_*s*_ and *CF* ≈ *ϕEF*.

## 3 Results

For presentation purposes only figures for the municipalities af Barranquilla (8001), Giron (68307), Monteria (23001), Santa Marta (47001), Sincelejo (70001), Ibague (73001), Cali (76001), and Arauca (81001) are shown. These were empirically selected as a representative sub-sample by distribution of population sizes. The complete set of plots can be found in the S1 Appendix.

### 3.1 Estimated parameters for ZIKV infection

Using the best-fit parameters and correction factor from the Monte Carlo procedure, the number of weekly ZIKV infections from epidemiological week 1, 2015 through 52, 2017 was estimated. Infections were disaggregated by sex, symptomatology, and transmission pathway. The expansion factor was calculated for the symptomatic infections (*EF*_*s*_) and for the total number of infections (*EF*) are shown in Table 3, which also shows the estimated parameters and correction factor for the symptomatic infections in the 27 considered municipalities. The root-mean-square error (RMSE) of the compartmental model is also reported.

**Table 3:** Estimated errors, parameters, and correction factor for the symptomatic ZIKV infections.

Code	Municipality	*CF*	*RMSE*	*β*_*vh*_	*β*_*hv*_	*β*_*wm*_	*β*_*mw*_	*ϕ*	*γ*_2_	*γ*_3_
8001	Barranquilla	7.4	0.00	0.1	0.4	0.03	0.05	0.16	0.09	0.13
8758	Soledad	20.60	0.00	0.23	0.16	0.01	0.04	0.16	0.08	0.39
13001	Cartagena	20.90	0.00	0.19	0.23	0.03	0.05	0.16	0.06	0.49
18001	Florencia	4.40	0.00	0.26	0.15	0.02	0.08	0.16	0.07	0.39
23001	Monteria	9.30	0.00	0.17	0.40	0.03	0.10	0.16	0.10	0.14
41001	Neiva	1.90	0.00	0.22	0.36	0.02	0.10	0.16	0.09	0.15
41298	Garzon	2.50	0.00	0.16	0.28	0.02	0.09	0.16	0.06	0.20
47001	Santa Marta	9.20	0.00	0.25	0.15	0.03	0.05	0.16	0.09	0.11
50001	Villavicencio	2.90	0.00	0.28	0.11	0.01	0.05	0.16	0.06	0.14
50006	Acacias	2.40	0.00	0.19	0.27	0.02	0.04	0.16	0.09	0.46
54001	Cucuta	2.10	0.00	0.18	0.30	0.00	0.02	0.16	0.09	0.23
54405	Los Patios	1.90	0.00	0.30	0.39	0.02	0.07	0.16	0.09	0.34
54874	Villa del Rosario	1.60	0.00	0.13	0.33	0.03	0.05	0.16	0.10	0.37
68001	Bucaramanga	1.20	0.00	0.23	0.13	0.01	0.06	0.16	0.06	0.11
68081	Barrancabermeja	4.40	0.00	0.28	0.09	0.01	0.07	0.16	0.09	0.48
68276	Floridablanca	1.20	0.00	0.26	0.14	0.02	0.04	0.16	0.06	0.18
68307	Giron	1.90	0.00	0.26	0.11	0.00	0.06	0.16	0.08	0.16
68547	Piedecuesta	2.30	0.00	0.15	0.20	0.03	0.01	0.16	0.06	0.13
70001	Sincelejo	13.70	0.00	0.10	0.40	0.03	0.10	0.16	0.10	0.12
73001	Ibague	4.60	0.00	0.12	0.32	0.01	0.09	0.16	0.06	0.11
76001	Cali	2.00	0.00	0.09	0.32	0.02	0.02	0.21	0.08	0.36
76111	Guadalajara de Buga	1.10	0.00	0.19	0.28	0.02	0.02	0.17	0.07	0.39
76147	Cartago	1.80	0.00	0.22	0.18	0.01	0.03	0.16	0.07	0.26
76520	Palmira	2.10	0.00	0.15	0.23	0.02	0.04	0.16	0.08	0.17
81001	Arauca	4.00	0.00	0.24	0.12	0.03	0.07	0.16	0.06	0.17
85001	Yopal	3.40	0.00	0.12	0.30	0.02	0.02	0.16	0.07	0.30
88001	San Andres	2.30	0.00	0.28	0.31	0.01	0.08	0.16	0.06	0.28

Among the municipalities, Soledad, Florencia, Neiva, Santa Marta, Villavicencio, Los Patios, Bucaramanga, Barrancabermeja, Floridablanca, Giron, Cartago, and San Andres exhibited relatively high vector-human transmission probabilities (*β*_*vh*_ > 0.20). These estimates imply that the rapid increase in the number of ZIKV infections may be related to the short assumed latent periods. Interestingly, neither the female-to-male (*β*_*wm*_) or male-to-female (*β*_*mw*_) transmission probabilities exhibited any particularly obvious pattern or bias to specific values. These sexual transmission probabilities tend to be away from their minimum value though, implying that the pathways are not insignificant. No significant correlations were identified between sexual transmission and external variables such as population size.

**Fig 2:**
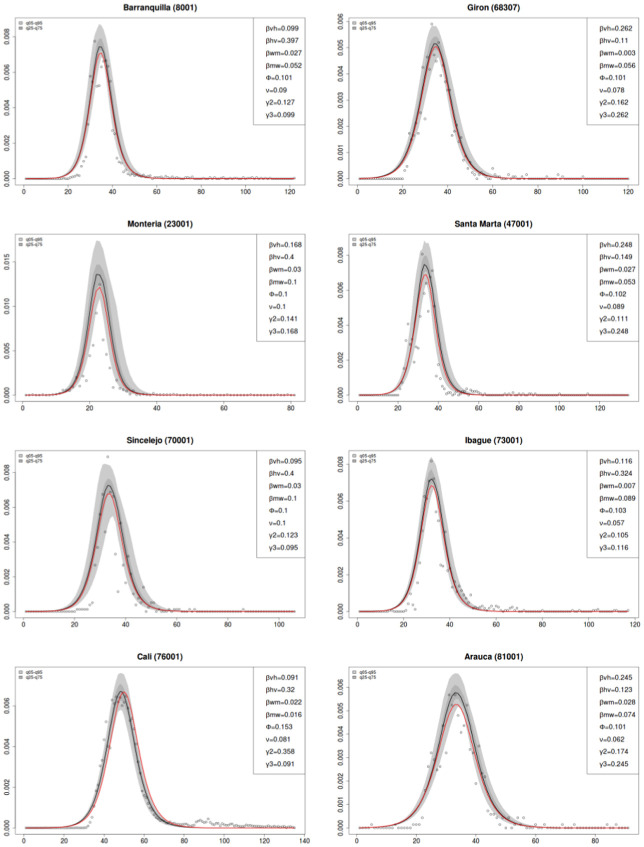
Comparing the adjusted proportion of observed symptomatic infections and the model output (See S1 Appendix for all plots). Y-axis is a proportion (infected / at risk population), and the x-axis is time (in weeks).

Fig 2 shows a subset of municipalities over the two-year period considered, adjusted by *CF*, and compared to the proportion of symptomatic infections estimated using the mathematical model proposed herein. The aforementioned MCMC implementation was utilized to approximate sensitivity of the model to slight parameter changes, with a measure of confidence in the estimated model highlighted (see S1 Appendix for complete list of plots). A direct implementation of the compartmental model is included and depicted in each plot as a red line.

### 3.2 Reporting degree

Table 4 compares the number of reported infections to the number of estimated infections, along with the correction and expansion factors for the symptomatic infections (*EF*_*s*_) and the total number of infections (*EF*). The estimates of *EF*_*s*_ ranged from 0.95 to 25.63, with the highest underreporting degree in Cartagena and the smallest in Guadalajara de Buga. The estimates of *EF* ranged from 6.76 to 195.66, with Guadalajara de Buga as the municipality with the smallest overall underreporting degree and Cartagena the municipality with the highest. Guadalajara de Buga is the only municipality having *EF*_*s*_ < 1, but all expansion factors are greater than one. The CF and *Ef*_*s*_ factors tend to have a small raw difference in value, implying that the fitted model may well-represent the observed data. Symptomatic expansion factors (*EF*_*s*_) for Bucaramagna, Floridablanca, and Guadalajara de Buga have their *Ef*_*s*_±0.25, which shows a likely good surveillance process and/or high reporting rate. Using the model, we see that a under-reporting of symptomatic infections is a common occurrence, as is now well known. The lowest (*EF* = 159.20) and greatest (*EF* = 5.58) overall reporting degree were observed for Cartagena and Guadalajara de Buga, where 0.6% and 17.9% of overall ZIKV infections were estimated to have been reported, respectively. The high overall expansion factors were expected given that the proportion of symptomatic infections (*ϕ*) for most all of the municipalities was estimated to be at the lower permitted limit. It should be recognized that most of the highest expansion factor municipalities, e.g., Soledad, Cartagena, Monteria, Sincelejo, are high population areas but that population alone is insufficient to characterize the calculated EF. For instance, Cali is a high population municipality with low EF, but partly perhaps the difference could be partly attributed to it being approximately 1000m above sea level. Further discussion about this issue will be in Section 4.

**Table 4:** Comparison between the number of reported infections and estimated infections.

Code	Municipality	Reported infections	Estimated infections		Expansion factor		
			Symptomatic	Total	*CF*	*Ef*_*s*_	EF
8001	Barranquilla	2294	19534	121700	7.40	8.52	53.05
8758	Soledad	513	12157	75428	20.60	23.70	147.03
13001	Cartagena	921	23607	146627	20.90	25.63	159.20
18001	Florencia	542	3517	21855	4.40	6.49	40.32
23001	Monteria	1208	17980	112383	9.30	14.88	93.03
41001	Neiva	2268	5733	35735	1.90	2.53	15.76
41298	Garzon	723	2068	12825	2.50	2.86	17.74
47001	Santa Marta	1101	11997	74157	9.20	10.90	67.35
50001	Villavicencio	2061	6467	40247	2.90	3.14	19.53
50006	Acacias	511	1291	8053	2.40	2.53	15.76
54001	Cucuta	5462	14028	87462	2.10	2.57	16.01
54405	Los Patios	736	1346	8404	1.90	1.83	11.42
54874	Villa del Rosario	659	1650	10223	1.60	2.50	15.51
68001	Bucaramanga	4282	5350	33225	1.20	1.25	7.76
68081	Barrancabermeja	729	3496	21826	4.40	4.80	29.94
68276	Floridablanca	1872	2202	13738	1.20	1.18	7.34
68307	Giron	1019	2100	13080	1.90	2.06	12.84
68547	Piedecuesta	595	1485	9247	2.30	2.50	15.54
70001	Sincelejo	572	9902	61890	13.70	17.31	108.20
73001	Ibague	1386	7250	44578	4.60	5.23	32.16
76001	Cali	16053	31544	147798	2.00	1.96	9.21
76111	Guadalajara de Buga	1391	1328	7758	1.10	0.95	5.58
76147	Cartago	915	1949	11941	1.80	2.13	13.05
76520	Palmira	1524	3429	21131	2.10	2.25	13.87
81001	Arauca	557	2438	15178	4.00	4.38	27.25
85001	Yopal	690	2798	17326	3.40	4.06	25.11
88001	San Andres	1088	2999	18312	2.30	2.76	16.83

The proportion of symptomatic infections reported is smaller than 50% in municipalities except Los Patios (55%), Bucaramanga (80%), Floridablanca (85%), Cali (51%) and Guadalajara de Buga. Our estimates imply significant disparities in the rates of reporting across the municipalities and in general very low reporting rates reiterating the mildness of ZIKV symptoms.

The variation of the parameters and the expansion factor between different regions can be attributed to distinct geographic and socioeconomic conditions that favor transmission and might allow for superspreading events to occur. In these events, a small proportion of the population is responsible for a large proportion of cases. On the one hand, Colombia, being one of the countries that lie near the equator, has a very diverse climate that varies with the elevation and is more or less constant throughout the year. Thus, regions in lower elevations are much more suitable for mosquito breeding. On the other hand, the living conditions and the lack of access to water and sewage systems make some populations more likely to be exposed to mosquito clusters than others.

### 3.3 ZIKV infections disaggregated by symptomatology

Table 5 shows the total number of ZIKV infections estimated by municipality, along with the percentage of symptomatic and asymptomatic cases, disaggregated by sex. Given the generally low reporting rate (symbol *ϕ*) for each municipality model, it is expected that the numbers appear somewhat homogeneous across municipalities. The exception being Cali, where approximately 21% of individuals were symptomatic. In general, females were slightly more represented than males, which would follow literature such as [111], in that the concern about pregnancy might have introduced referral and testing bias, which led to a report where only 33.33% of the ZIKV infections correspond to males. This is also explainable by a number of reasons. First, the proportion of women is greater than the proportion of men in all the municipalities, aside from Garzon, Yopal, and Acacias. Second, the transmission rate from men to women (*β*_*mw*_) is equal or greater than the transmission rate from women to men (*β*_*wm*_).

**Table 5:** ZIKV infections disaggregated by symptomatology.

Code	Municipality	Estimated infections	Symptomatic (%)			Asymptomatic (%)		
			Women	Men	Total	Women	Men	Total
8001	Barranquilla	121700	8.23	7.82	16.05	43.02	40.93	83.95
8758	Soledad	75428	8.14	7.98	16.12	42.36	41.52	83.88
13001	Cartagena	146627	8.29	7.81	16.10	43.20	40.70	83.90
18001	Florencia	21855	8.19	7.91	16.09	42.68	41.23	83.91
23001	Monteria	112383	8.23	7.77	16.00	43.19	40.81	84.00
41001	Neiva	35735	8.36	7.68	16.04	43.75	40.21	83.96
41298	Garzon	12825	7.74	8.38	16.12	40.27	43.60	83.88
47001	Santa Marta	74157	8.25	7.92	16.18	42.76	41.06	83.82
50001	Villavicencio	40247	8.23	7.83	16.07	43.01	40.92	83.93
50006	Acacias	8053	7.97	8.06	16.03	41.74	42.23	83.97
54001	Cucuta	87462	8.26	7.78	16.04	43.23	40.73	83.96
54405	Los Patios	8404	8.32	7.70	16.02	43.62	40.36	83.98
54874	Villa del Rosario	10223	8.15	7.99	16.14	42.34	41.52	83.86
68001	Bucaramanga	33225	8.31	7.79	16.10	43.30	40.60	83.90
68081	Barrancabermeja	21826	8.13	7.89	16.02	42.61	41.37	83.98
68276	Floridablanca	13738	8.34	7.69	16.03	43.71	40.26	83.97
68307	Giron	13080	8.08	7.97	16.06	42.26	41.68	83.94
68547	Piedecuesta	9247	8.19	7.87	16.06	42.78	41.16	83.94
70001	Sincelejo	61890	8.14	7.86	16.00	42.72	41.28	84.00
73001	Ibague	44578	8.36	7.91	16.26	43.03	40.71	83.74
76001	Cali	147798	10.99	10.36	21.34	40.49	38.17	78.66
76111	Guadalajara de Buga	7758	8.71	8.40	17.12	42.20	40.68	82.88
76147	Cartago	11941	8.42	7.90	16.32	43.20	40.48	83.68
76520	Palmira	21131	8.34	7.89	16.23	43.04	40.73	83.77
81001	Arauca	15178	8.11	7.95	16.06	42.38	41.56	83.94
85001	Yopal	17326	8.07	8.08	16.15	41.89	41.96	83.85
88001	San Andres	18312	8.24	8.14	16.38	42.05	41.57	83.62

### 3.4 ZIKV infections disaggregated by transmission pathway

Table 6 shows the total number of ZIKV infections estimated by municipality, along with the percentage of horizontal (sexually acquired) and vector-borne infections. The percentages of horizontal and vector-borne infections are also disaggregated by sex. According to these estimates, the proportion of sexually transmitted ZIKV infections can be as low as 0.5% and as high as 2.5%. These estimates are lower than the values presented in [59]: 4.44% (95% CI: 0.297%-23.02%), however, recent findings [94] imply that our lower estimate may be more realistic, especially given that a mosquito infection may be more likely to be first acquired. Although sexual transmission constitutes a small percentage of the total infections, the total number of cases is significant. The smallest proportion of horizontal infections was found in Cucuta, where 296 infections were sexually acquired. The largest proportion corresponds to Sincelejo, where 2974 infections were a result of the sexual transmission.

**Table 6:** ZIKV infections disaggregated by transmission pathway.

Code	Municipality	Estimated infections	Symptomatic (%)			Asymptomatic (%)		
			Women	Men	Total	Women	Men	Total
8001	Barranquilla	121700	1.03	0.42	1.45	41.04	37.37	78.41
8758	Soledad	75428	0.96	0.14	1.10	39.65	38.89	78.54
13001	Cartagena	146627	1.08	0.24	1.33	41.25	37.14	78.40
18001	Florencia	21855	1.75	0.20	1.95	39.43	38.30	77.73
23001	Monteria	112383	1.39	0.35	1.74	40.86	37.40	78.26
41001	Neiva	35735	1.37	0.21	1.58	41.92	36.40	78.32
41298	Garzon	12825	1.62	0.24	1.86	34.89	43.14	78.04
47001	Santa Marta	74157	1.03	0.45	1.48	40.43	37.58	78.00
50001	Villavicencio	40247	1.38	0.26	1.64	40.65	37.49	78.14
50006	Acacias	8053	0.68	0.15	0.83	38.74	40.30	79.04
54001	Cucuta	87462	0.32	0.01	0.34	42.13	37.47	79.60
54405	Los Patios	8404	0.73	0.10	0.82	42.35	36.83	79.18
54874	Villa del Rosario	10223	0.94	0.27	1.21	39.62	38.72	78.33
68001	Bucaramanga	33225	1.63	0.23	1.86	40.96	36.86	77.82
68081	Barrancabermeja	21826	1.60	0.17	1.77	39.46	38.69	78.15
68276	Floridablanca	13738	1.20	0.23	1.43	42.31	36.24	78.54
68307	Giron	13080	1.36	0.05	1.41	38.95	39.46	78.41
68547	Piedecuesta	9247	0.42	0.50	0.92	41.33	37.60	78.93
70001	Sincelejo	61890	1.85	0.50	2.35	39.49	38.13	77.63
73001	Ibague	44578	2.14	0.11	2.25	39.61	37.30	76.91
76001	Cali	147798	0.36	0.22	0.58	36.98	32.07	69.05
76111	Guadalajara de Buga	7758	0.39	0.14	0.53	39.57	36.88	76.45
76147	Cartago	11941	0.59	0.04	0.64	41.73	36.73	78.46
76520	Palmira	21131	0.94	0.26	1.19	41.12	37.03	78.16
81001	Arauca	15178	1.83	0.40	2.23	38.70	38.86	77.56
85001	Yopal	17326	0.42	0.29	0.71	39.26	39.58	78.84
88001	San Andres	18312	1.04	0.07	1.11	38.84	38.94	77.77

### 3.5 Implementation of public health policies

One important use of disease spread models is to inform potential public policy decisions. To this end we simulated three hypothetical scenarios where a set of health policies are applied at specific points during the outbreak and assess the impact of the policy for mitigating ZIKV spread. These are not intended to be exhaustive, nor hyper-realistic. Rather, to further the discussion concerning uses of the proposed model.

**Fig 3:**
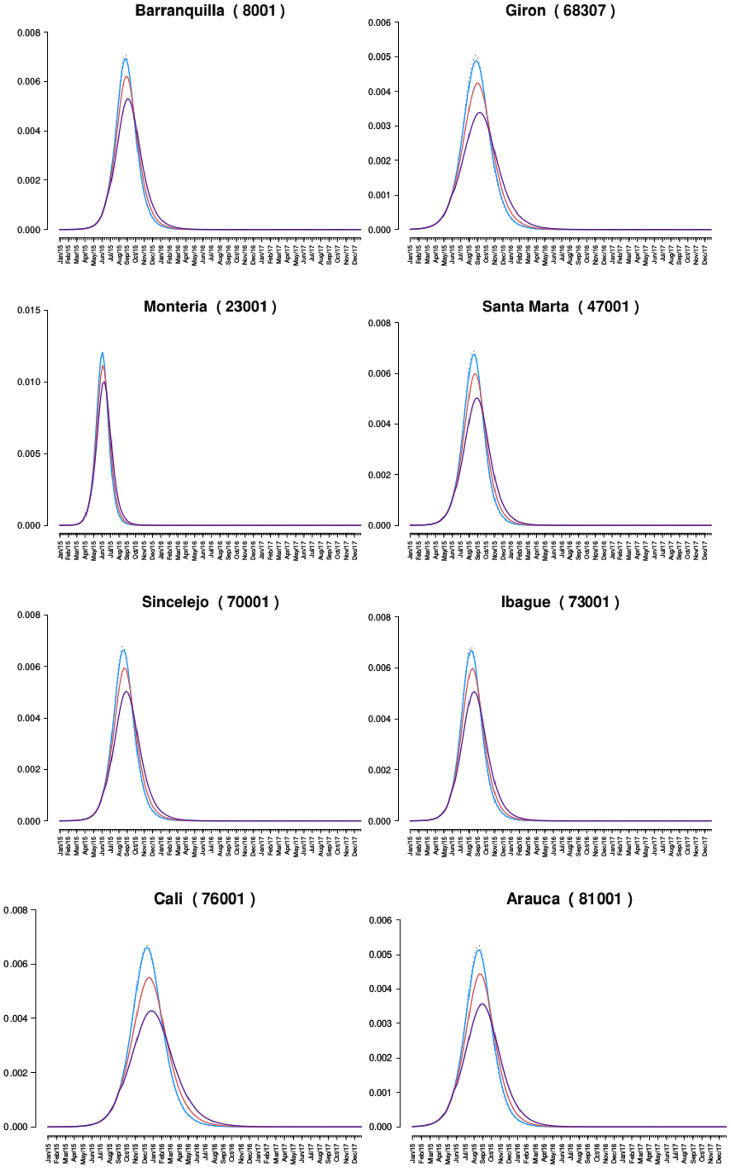
Comparison of the proportion of symptomatic infections of the best fit and the behavior of the epidemics when policies are implemented at th = 20%. Y-axis is a proportion (infected / at risk population), and the x-axis is time (in weeks). Best fit is dashed line, and policy 1,2, and 3 are colored blue, red, and purple, respectively.

**Fig 4:**
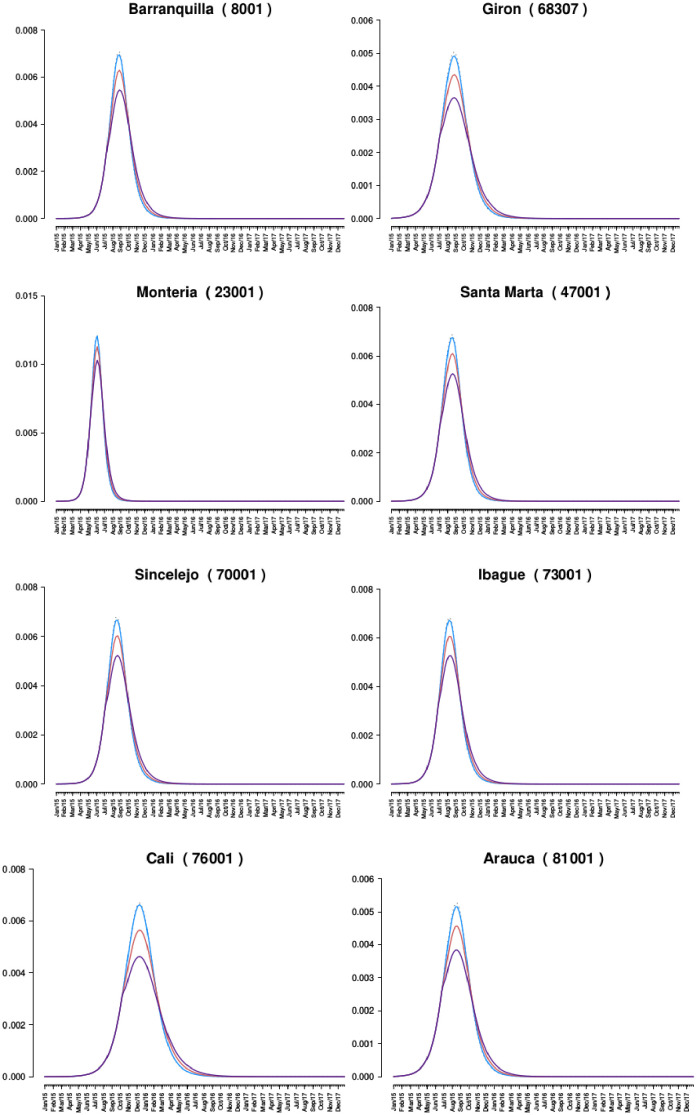
Comparison of the proportion of symptomatic infections of the best fit and the behavior of the epidemics when policies are implemented at th = 50%. Y-axis is a proportion (infected / at risk population), and the x-axis is time (in weeks). Best fit is dashed line, and policy 1,2, and 3 are colored blue, red, and purple, respectively.

**Fig 5:**
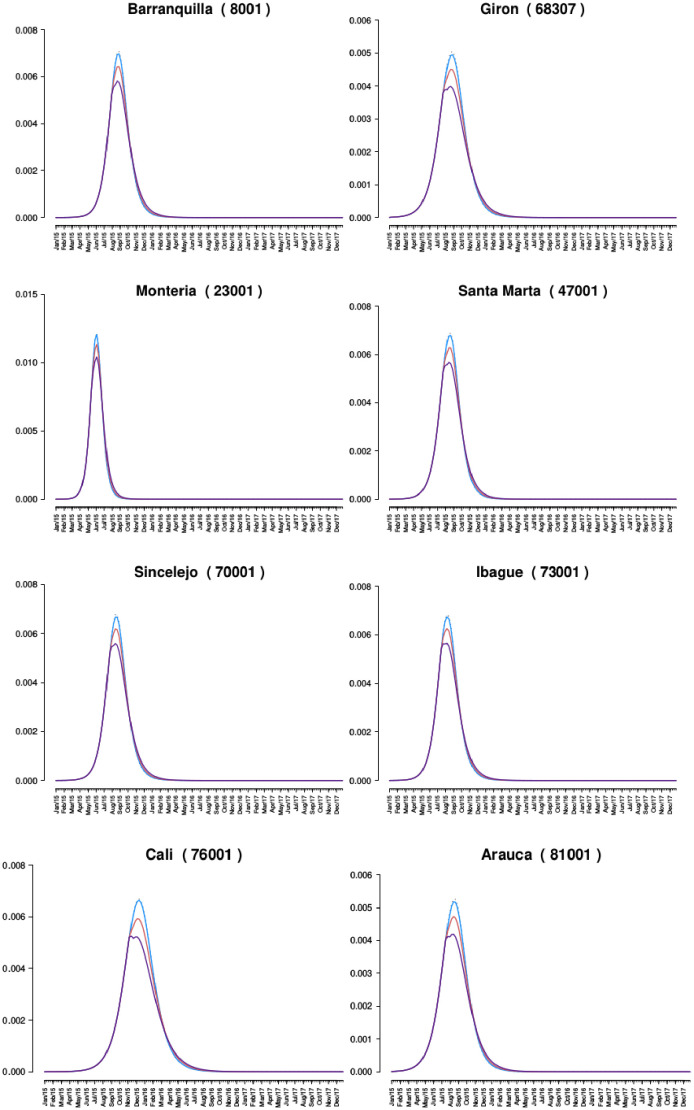
Comparison of the proportion of symptomatic infections of the best fit and the behavior of the epidemics when policies are implemented at th = 80%. Y-axis is a proportion (infected / at risk population), and the x-axis is time (in weeks). Best fit is dashed line, and policy 1,2, and 3 are colored blue, red, and purple, respectively.

The best-fit model for each municipality was used as a null model. Three time points were selected as the point where an intervention would occur by public policy, intended to coincide with levels of aggressiveness with respect to policy implementation: *(a) Threshold(th) = 20%:* when there were at least 20% of the maximum number of cases per week for the first time (Fig 3), *(b) Threshold(th) = 50%:* when there were at least 50% of the maximum number of cases per week for the first time (Fig 4), and *(c) Threshold(th) = 80%:* when there were at least 80% of the maximum number of cases per week for the first time (Fig 5).

1. **Policy one:** Promoting the use of condoms during sexual encounters regardless of the marital status and the use of other non-barrier contraception methods. *Scenario simulated:* The proportion of risky sexual encounters (rho) decreases by 50%.
2. **Policy two:** Promoting the use of insecticides and the destruction of larval breeding sites using public broadcasting. *Scenario simulated:* The population of mosquitoes is reduced by 10%.
3. **Policy three:** Promoting the use of insecticides and the destruction of larval breeding sites more extensively, using public broadcasting and making visits to areas where poverty limits or restring the access to information media. *Scenario simulated:* The population of mosquitoes is reduced by 20%.

Table 7 shows the number of symptomatic and asymptomatic ZIKV infections that occurred during the outbreak null model (best-fit model output without policies) and the percentage reduction that each policy provides when applied at the three different above-stated points of the outbreak. The policies that implement vector control are more effective than the policy that promotes safe sexual practices, as expected. At th = 20%, policy two prevents typically 4–10 times as many infections across the 27 municipalities as policy one. Policy 3 prevents approximately 20–30 times as many cases as policy one, and 2.0–2.5 times as many cases as policy two. Similar proportions are observed for th = 50% and th = 80%.

**Table 7:** Percentage of reduction of ZIKV infections by policy.

Code	Municipality	Infections	th = 20%			th = 50%			th = 80%		
			Policy 1	Policy 2	Policy 3	Policy 1	Policy 2	Policy 3	Policy 1	Policy 2	Policy 3
8001	Barranquilla	121721	0.27	2.46	5.88	0.26	2.36	5.59	0.24	2.25	5.26
8758	Soledad	74885	0.33	2.51	6.04	0.31	2.41	5.74	0.28	2.22	5.20
13001	Cartagena	146565	0.17	1.81	4.44	0.16	1.74	4.24	0.14	1.59	3.79
18001	Florencia	21616	0.24	2.41	5.85	0.23	2.32	5.58	0.20	2.13	5.03
23001	Monteria	112385	0.04	0.63	1.60	0.04	0.62	1.56	0.04	0.59	1.45
41001	Neiva	35659	0.02	0.39	1.00	0.02	0.38	0.98	0.02	0.36	0.91
41298	Garzon	12730	0.11	1.66	4.02	0.10	1.61	3.86	0.10	1.53	3.62
47001	Santa Marta	73505	0.29	2.71	6.57	0.27	2.56	6.11	0.25	2.41	5.67
50001	Villavicencio	39999	0.63	4.60	10.94	0.60	4.38	10.30	0.52	3.92	9.00
50006	Acacias	8011	0.10	1.40	3.47	0.09	1.34	3.29	0.09	1.26	3.04
54001	Cucuta	87314	0.15	1.09	2.69	0.15	1.07	2.62	0.14	1.02	2.48
54405	Los Patios	8399	0.00	0.07	0.21	0.00	0.07	0.21	0.00	0.07	0.20
54874	Villa del Rosario	10123	0.26	2.10	5.03	0.25	2.01	4.79	0.23	1.92	4.50
68001	Bucaramanga	32873	0.63	4.53	10.79	0.59	4.32	10.15	0.52	3.86	8.86
68081	Barrancabermeja	21276	1.12	7.18	16.89	1.04	6.71	15.46	0.90	5.86	13.08
68276	Floridablanca	13662	0.32	2.93	7.09	0.30	2.79	6.67	0.27	2.55	5.98
68307	Giron	13122	0.59	4.52	10.73	0.55	4.25	9.96	0.50	3.93	9.02
68547	Piedecuesta	9095	0.68	4.86	11.57	0.64	4.59	10.77	0.56	4.11	9.40
70001	Sincelejo	61892	0.28	2.65	6.31	0.27	2.57	6.08	0.25	2.40	5.59
73001	Ibague	44024	0.27	2.63	6.28	0.26	2.54	6.00	0.24	2.36	5.48
76001	Cali	154490	0.28	5.48	12.95	0.27	5.21	12.14	0.24	4.74	10.80
76111	Guadalajara de Buga	8000	0.07	1.01	2.54	0.06	0.97	2.40	0.06	0.90	2.19
76147	Cartago	11755	0.32	2.28	5.44	0.31	2.19	5.18	0.29	2.09	4.89
76520	Palmira	20685	0.44	3.63	8.70	0.41	3.45	8.19	0.37	3.18	7.41
81001	Arauca	15131	0.50	4.30	10.28	0.46	4.03	9.46	0.41	3.68	8.46
85001	Yopal	17089	0.40	3.05	7.24	0.39	2.95	6.94	0.36	2.76	6.40
88001	San Andres	18041	0.01	0.26	0.68	0.01	0.25	0.67	0.01	0.25	0.65

### 3.6 Variation of the number of risky sexual interactions

**Fig 6:**
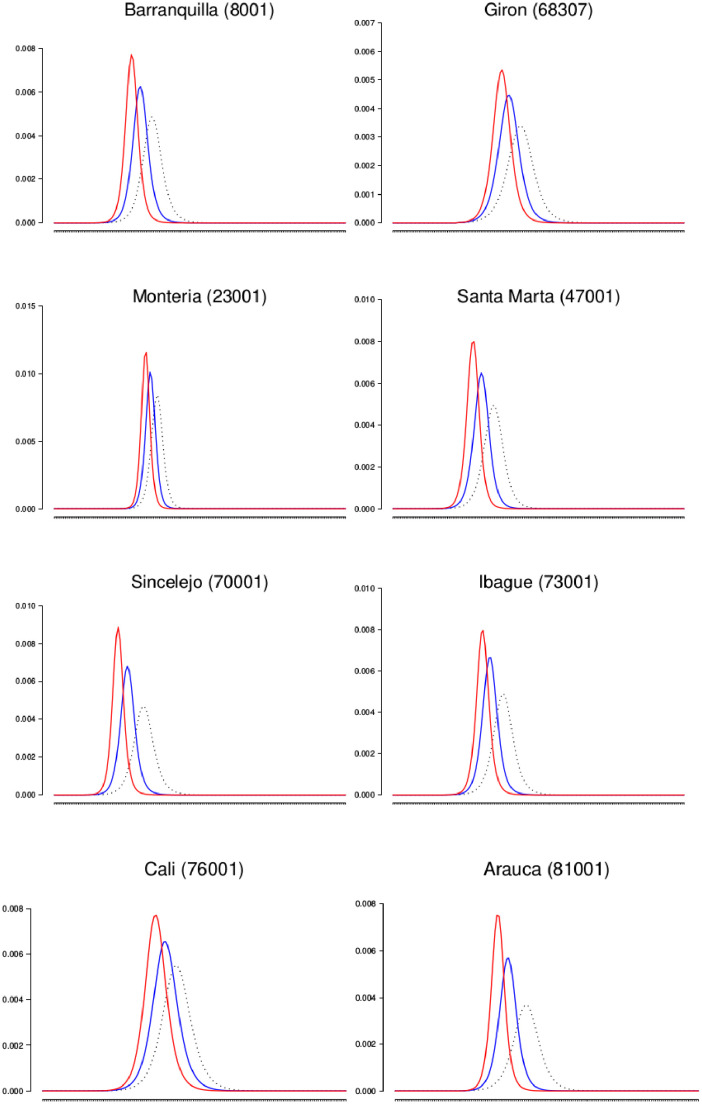
Comparison of the proportion of symptomatic infections of the best fit and the behavior of the epidemics when the number of risky sexual interactions increases. Y-axis is a proportion (infected / at risk population), and the x-axis is time (in weeks). Best fit is dashed line, blue corresponds to *ρ* = 0.10, and red is *ρ* = 0.20.

We also explored two feasible scenarios where the proportion of sexual interaction without the use of sexual protection (e.g., condoms) increases. On the one hand, we explore the case in which the number of sexual encounters per week increases resulting in a proportion of unsafe sexual interactions of 10%. Secondly, we additionally assume that individuals have two sexual partners per year, then rho increases to 20%. The second scenario will also occur if the number of sexual encounters per week increases. Fig 6 shows the comparison between the best fit results and the output of the model when the proportion of unprotected sexual interaction increases and shows significant increases if *ρ* ≥ 0.2, and for example Sincelejo (70001) and Arauca (81001) see nearly doubles proportions.

Table 8 shows the number of symptomatic and asymptomatic ZIKV infections that occurred during the outbreak and the percentage of new ZIKV infections when the proportion of risky sexual interactions increases. If the proportion of risky sexual interactions increases to 10% there would be additional infections. In the case that rho increases to 20% there would be an increment of 80,479 infections, with respect to the value used to fit the model. The results of these simulations show that a slightly higher proportion of unprotected sexual interactions, given either by the increase of the number of sexual partners per year or by the frequency of sexual encounters, might lead to a significant number of additional ZIKV infections. Therefore, although horizontal transmission is not the main pathway of Zika, the implemented policies should also promote the use of condoms or other forms of sexual protection as a safe sexual practice that minimizes the acquisition of sexual infections and, in the case of ZIKV, reduces the propagation of the virus.

**Table 8:** Percentage of increment of ZIKV infections according to the proportion of unprotected sexual interactions.

Code	Municipality	Infections	Increment (%)	
			*ρ* = 10%	*ρ* = 20%
8001	Barranquilla	121841	2.96	4.82
8758	Soledad	75626	2.16	3.44
13001	Cartagena	146952	1.84	2.82
18001	Florencia	21905	2.55	3.44
23001	Monteria	112385	0.61	0.88
41001	Neiva	35773	0.31	0.42
41298	Garzon	12862	1.65	2.28
47001	Santa Marta	74448	3.05	4.81
50001	Villavicencio	40313	5.82	8.76
50006	Acacias	8062	0.70	1.19
54001	Cucuta	87541	0.33	0.60
54405	Los Patios	8410	0.03	0.05
54874	Villa del Rosario	10256	1.84	3.03
68001	Bucaramanga	33303	6.06	8.70
68081	Barrancabermeja	21837	8.80	12.86
68276	Floridablanca	13749	3.19	4.81
68307	Giron	13101	5.00	7.36
68547	Piedecuesta	9261	3.74	6.62
70001	Sincelejo	61892	4.11	5.95
73001	Ibague	44832	3.71	4.65
76001	Cali	155347	3.36	6.23
76111	Guadalajara de Buga	7917	0.50	0.90
76147	Cartago	12024	1.56	2.61
76520	Palmira	21237	3.14	5.12
81001	Arauca	15202	6.81	9.41
85001	Yopal	17387	1.83	3.39
88001	San Andres	18459	0.11	0.14

## 4 Discussion

We presented a deterministic model for the Zika Virus spread as both a vector-borne and sexually transmitted disease. Our model encompasses transmission of the virus between humans and mosquitoes, and between women and men regardless of the presence or absence of symptoms. Our analysis disaggregated the estimated infections according to the sex, symptomatology and transmission pathway. We also evaluated the reporting degree for the symptomatic infections and the total ZIKV infections. While the proposed model has been demonstrated to well-fit the observed patterns for most of the municipalities, there are some important aspects to discuss concerning the model, its assumptions, and potential influential external factors.

Firstly, the proposed transmission model does not consider male-male, or female-female transmission, which although comprise a small proportion of all cases, does contribute to the spread dynamics. The model also does not consider environmental factors such as sea level that have been observed to be high related to ZIKV incidence, mostly by influencing the mosquito population (more below). Moreover, the model does not consider cross-immunity directly as it assumes the population of susceptible men and women is relatively well known, similar for the susceptible mosquito population. Due to the number of parameters the fitting procedure needed to be multiple-stages to avoid being trapped at local optimal that may not have practical relevance. Increased data or improved knowledge of the parameterization will reduce this issue. Additionally, the model was fit for each of the 27 municipalities independently, however, there are model parameters that should have similar values independent of the location such as the mean time to viral clearance in semen or vaginal fluid. Perhaps a more sophisticated higher-order model could be constructed and fit that is able to better make use of the data and represent such constraints.

The population at risk is estimated based on the average temperature and Index of Unsatisfied Basic Needs, which impacts the number of susceptible individuals and hence is an important factor to the overall disease spread. In this paper we assumed previous literature values, but calibrating this value is of critical importance. Of course, there are a variety of risk factors behind the geographical spread and transmission of ZIKV, for instance the inverse relationship between ZIKV and gross domestic product [112]. The mosquito population is also at risk and it is known that temperature also plays an important role [113, 114], and will vary between the affected municipalities. Temperature will vary through the year, and including this behavior into our model could improve the predictions further, especially for the few cases with very high correction factors.

The number of infections was adjusted using the a correction factor (the CF value) and their distribution in time by searching for the set of best fit parameters. The CF is applied uniformly to all time points, which is unlikely to be true in general, for instance as public awareness increases to attain more accurate reporting. Nevertheless, our results indicate significant levels of under-reporting in most municipalities seems likely. For example, the ZIKV infections reported in Monteria, Cucuta, Villa del Rosario and Florencia have a rapid increase, plausibly due to high levels of under-reporting at the beginning and at the end of the epidemic, respectively. On the other hand, the distribution of infections in Garzon and Acacias indicate a high level of under-reporting at the beginning of the outbreak only. The number of infections reported in Cartagena and Santamarta fluctuates over time, and could be due to inconsistent reporting of ZIKV infections throughout the epidemic. The results also suggest that some of the cases reported near the peak of infections in Neiva, Los Patios, Barrancabermeja, Guadalajara de Buga and San Andres, correspond to previous weeks. External factors, such as sea level, are likely also represented in this correction factor.

The data could be limited in a number of ways as well. The National System of Public Health Surveillance in Colombia (SIVIGILA) publishes time series of the number of symptomatic Zika infections that are confirmed clinically or through laboratory tests [65–67]. According to the procedure, serum samples have to be collected from symptomatic individuals with one of the following conditions: a) lives in or comes from areas in Colombia without verified transmission of the virus, b) comes from another country with or without Zika active circulation, c) belongs to an at-risk group. The samples are tested using molecular diagnostics based on RT-PCR [107, 108]. If the patient lives in or comes from areas with ongoing transmission of the virus and is exhibiting Zika-like symptoms that cannot be explained by other conditions, the cases are added to the surveillance system as clinically confirmed [107, 108]. Our model incorporates asymptomatic transmission of the ZIKV, which has proven to be significant [6] and we then estimated the proportion of symptomatic ZIKV infections reported (1/*EF*_*s*_) and the proportion of total ZIKV infections reported (1/*EF*). However, the restriction over laboratory testing may bias the results, as clinical testing relies on the symptomatology of the patient, the presence of similar viruses in the area, and the judgment of the doctor. Moreover, those who are tested must be sufficiently ill to visit the facility, but in many developing countries household economics may instead necessitate the individual to instead go to work.

We also estimated that the proportion of horizontal ZIKV infections in the municipalities studied was generally below 3%, which is inconsistent with the interval (4.44% (95% CI: 0.30%-23.02%)) presented in [59]. The sexual pathway of Zika has a small percentage contribution in the spread dynamics, however, these percentages are equivalent to a relevant number of infections that can be prevented through adequate sexual practices. It is important to note that these estimates were found considering that approximately 7% of the encounters were unprotected since the individuals involved were not incorporating sexual practices to protect themselves from sexually transmitted infections, including ZIKV. This proportion of risky interaction was calculated using the data from the Colombia Ministry of Health and Social Protection and Profamilia [95–97], which is not specific for each municipality. Specific factors such as income, level of education and access to medical care might expand or reduce this figure in a particular area. Detailed information concerning contraceptive use by municipality would allow us to improve our estimates for ZIKV horizontal transmission.

In cases where ZIKV is transmitted to a pregnant woman, her child may develop a variety of neurological disorders, such as GBS. In recording and comparing these numbers it is critical to be cognizant of potential false positives. For instance [115] report very high rates of false positives when testing for microcephaly (up to 82.5%) and GBS (up to 56%). These false-positives can have highly dramatic consequences, as indicated by a >90% increase in illegal abortion requests in Latin America during the 2016 epidemic [116]. Progress in the development of ZIKV diagnostic tests is outlined in [117], while the difficulties in obtaining reliable results have been discussed in [118–121]. Accurately detecting and estimating ZIKV cases is a major concern, but at present our proposed model does not consider such inconsistencies.

We also used the proposed model to provide some insight into the impact public policies could have on mitigating ZIKV spread. The experiments did not explicitly implement a policy, such as mosquito spraying, but instead simulated an estimate of what such a policy could mean with respect to changes in model parameterization. Given the relatively few cases of ZIKV due to sexual transmission, policies aimed at that particular pathway did not significantly impact overall spread. However, the associated risk of not putting resources toward mitigating sexual transmission is large given both potential immediate and long-term consequences and associated costs. In order to gain more granular insight the policies could be more concretely studied and related to specific value changes in parameterization. Moreover, the proposed model could instead form a basis for an network or agent-based simulation that would provide much more detailed feedback (e.g., [122]), but has very high computational burden and issues of calibration and relevance of output to the specifics “on the ground” is not clear. Moreover, the municipalities are considered independent entities, but in reality share common trade routes, transportation paths and may have many individuals commuting between them each day. Mitigating this level of interaction is not presently considered, but a patch model building from that proposed could provide detail on a larger scale than at a municipality level.

### 4.1 Conclusions

We constructed a compartmental model to represent the spread dynamics of ZIKV, including its two main transmission pathways: mosquito bites and sexual contact. The structure of our model allows for the estimation of cases by symptoms and sex. This is an important feature that can be utilized in the future to study specific populations, such as pregnant women who could be affected by the congenital diseases associated with ZIKV. We can also use it to study specific simulation scenarios, for example, the use of condoms as a health policy against the spread of Zika.

The ZIKV is a very atypical flavivirus, not only because of its causal association with severe disorders but also because of its multiple transmission pathways. The mathematical model we presented can be easily modified to add mechanisms that have not been explored, including non-sexual person-to-person transmission, which appears to be the pathway of the case reported in [14]. Nonetheless, the sexual transmission of the virus should not be neglected, as the fact that the Zika virus is transmissible from human to human implies a change in the global propagation dynamics. As humans act as vectors and reservoirs of the virus, the non-vector propagation is not affected by environmental conditions, such as seasonal change. This is a critical point that needs to be addressed in further research, in order to determine how the addition of these pathways impacts the global spread dynamics of the virus. Finally, considering that the ZIKV can be horizontal [123] and vertically [124, 125] transmitted from mosquito-to-mosquito, it would also be interesting to study the role of these interactions in the spread dynamics of the ZIKV. Certainly, the realization of these studies requires more information about the aforementioned mechanisms.

## Supporting information

S1 Appendix (PDF)
