# Supplementary material for: Modeling the spread of the Zika virus by sexual and mosquito transmission
Source: PLoS One. 2022 Dec 30;17(12):e0270127. doi: 10.1371/journal.pone.0270127 (PMC9803243; doi:10.1371/journal.pone.0270127)
Supplement: S1 Appendix — (PDF) [file pone.0270127.s001.pdf]

Appendix

5 Model fits

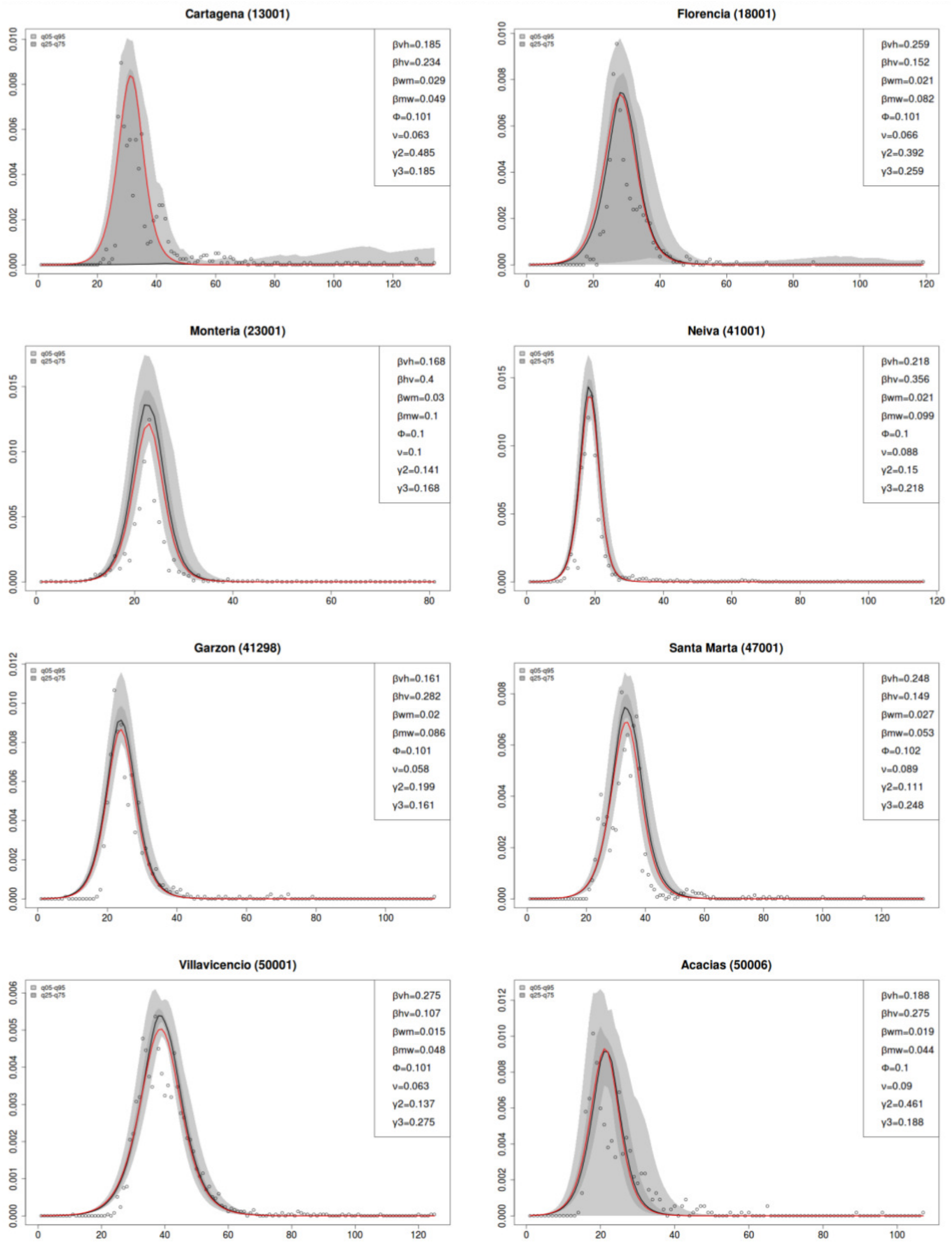

Figure 7. Comparing the adjusted proportion of observed symptomatic infections and the model output. Y-axis is a proportion (infected / at risk population), and the x-axis is time (in weeks).

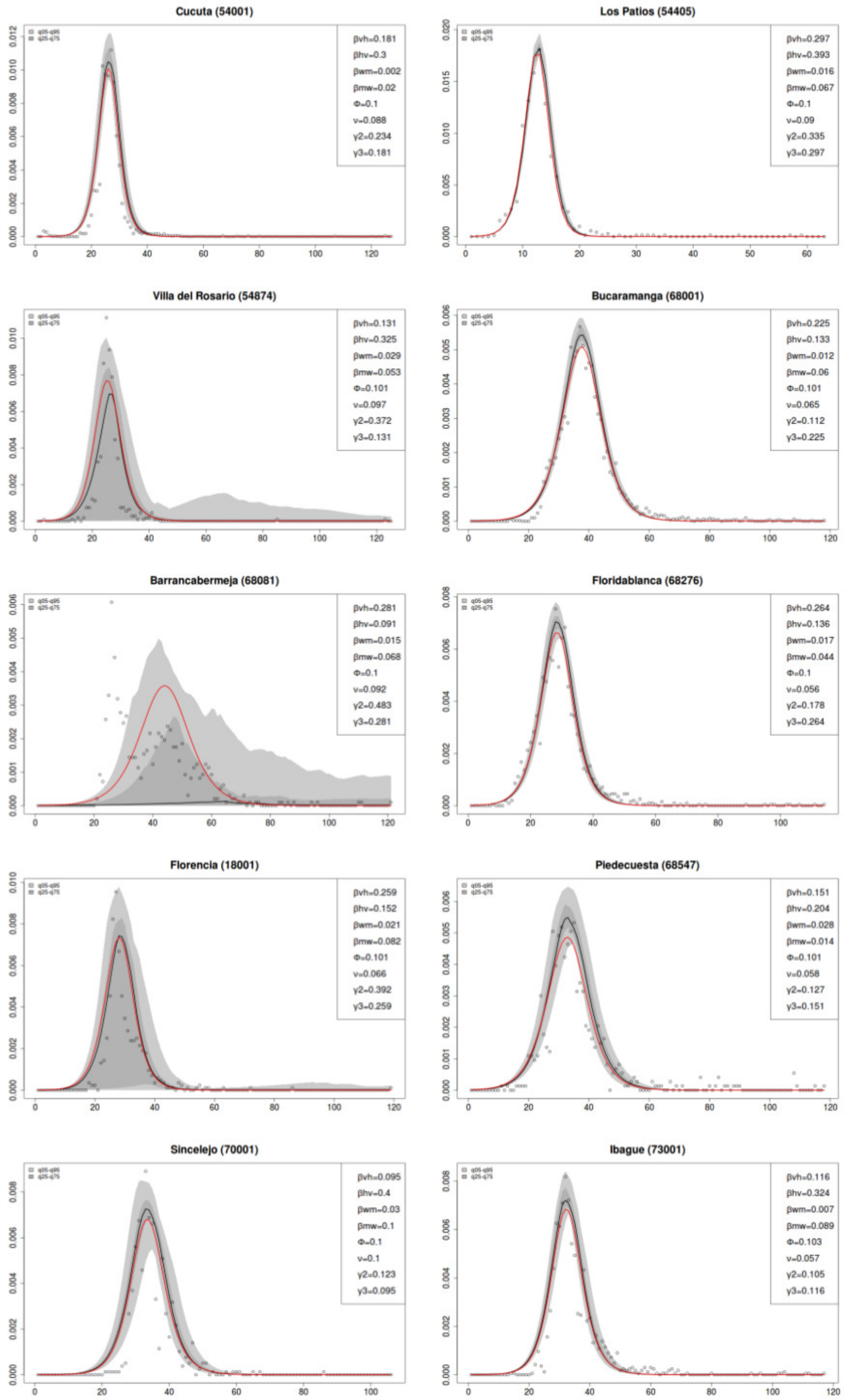

Figure 8. Comparing the adjusted proportion of observed symptomatic infections and the model output. Y-axis is a proportion (infected / at risk population), and the x-axis is time (in weeks).

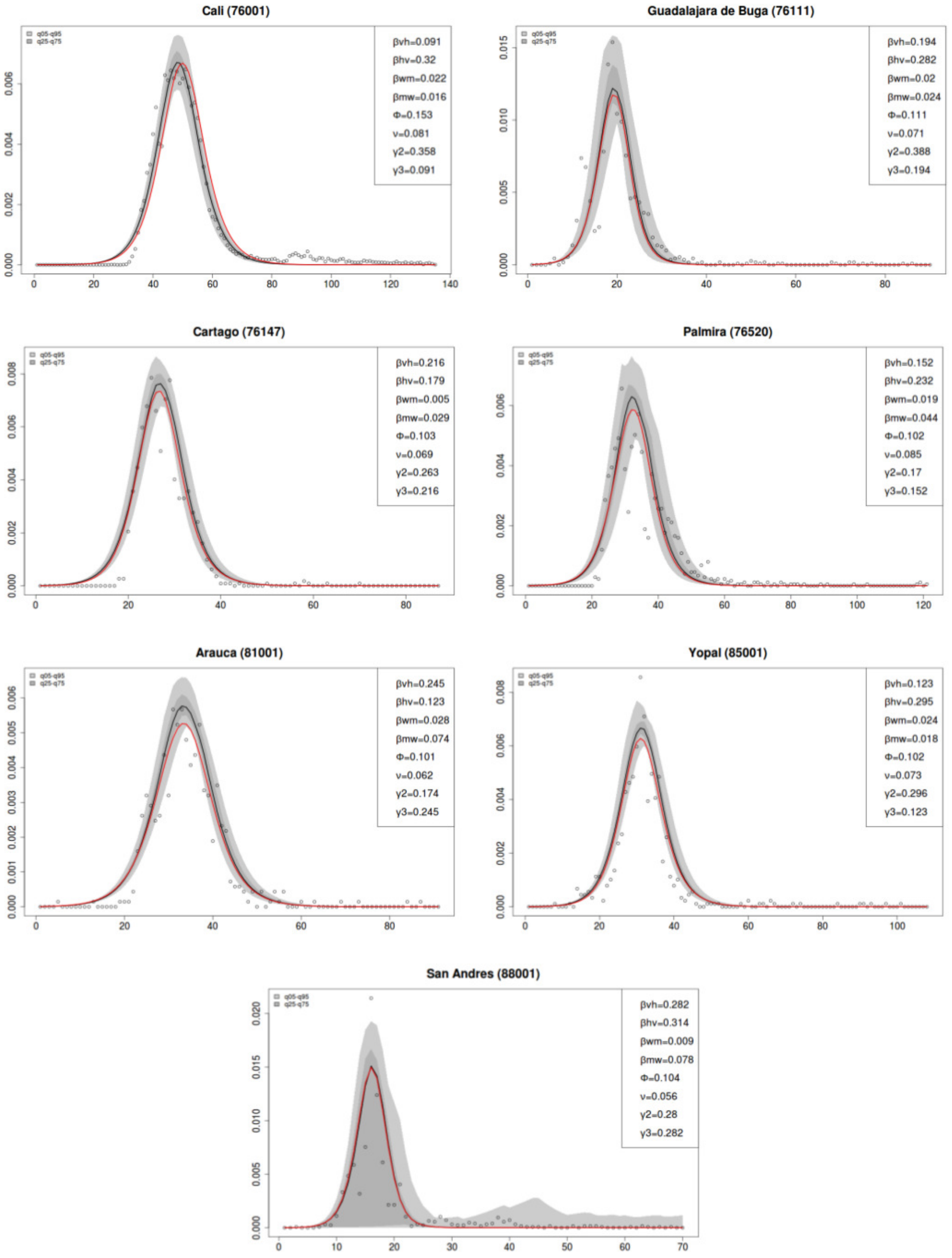

Figure 9. Comparing the adjusted proportion of observed symptomatic infections and the model output. Y-axis is a proportion (infected / at risk population), and the x-axis is time (in weeks).

6 Impact of Policies $th = 20\%$

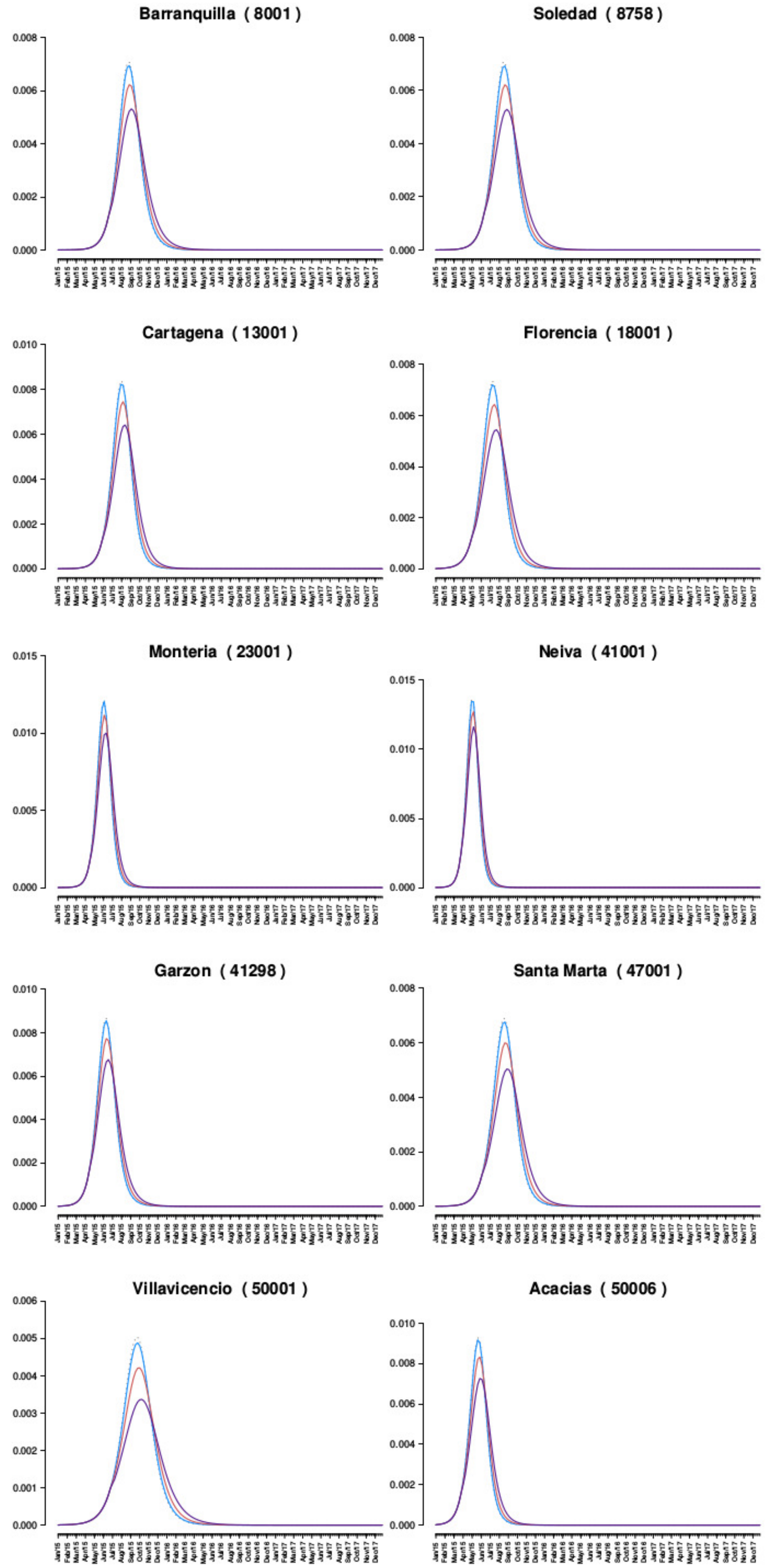

Figure 10. Comparison of the proportion of symptomatic infections of the best fit and the behavior of the epidemics when policies are implemented at th = 20%. Y-axis is a proportion (infected / at risk population), and the x-axis is time (in weeks). Best fit is dashed line, and policy 1,2, and 3 are colored blue, red, and purple, respectively.

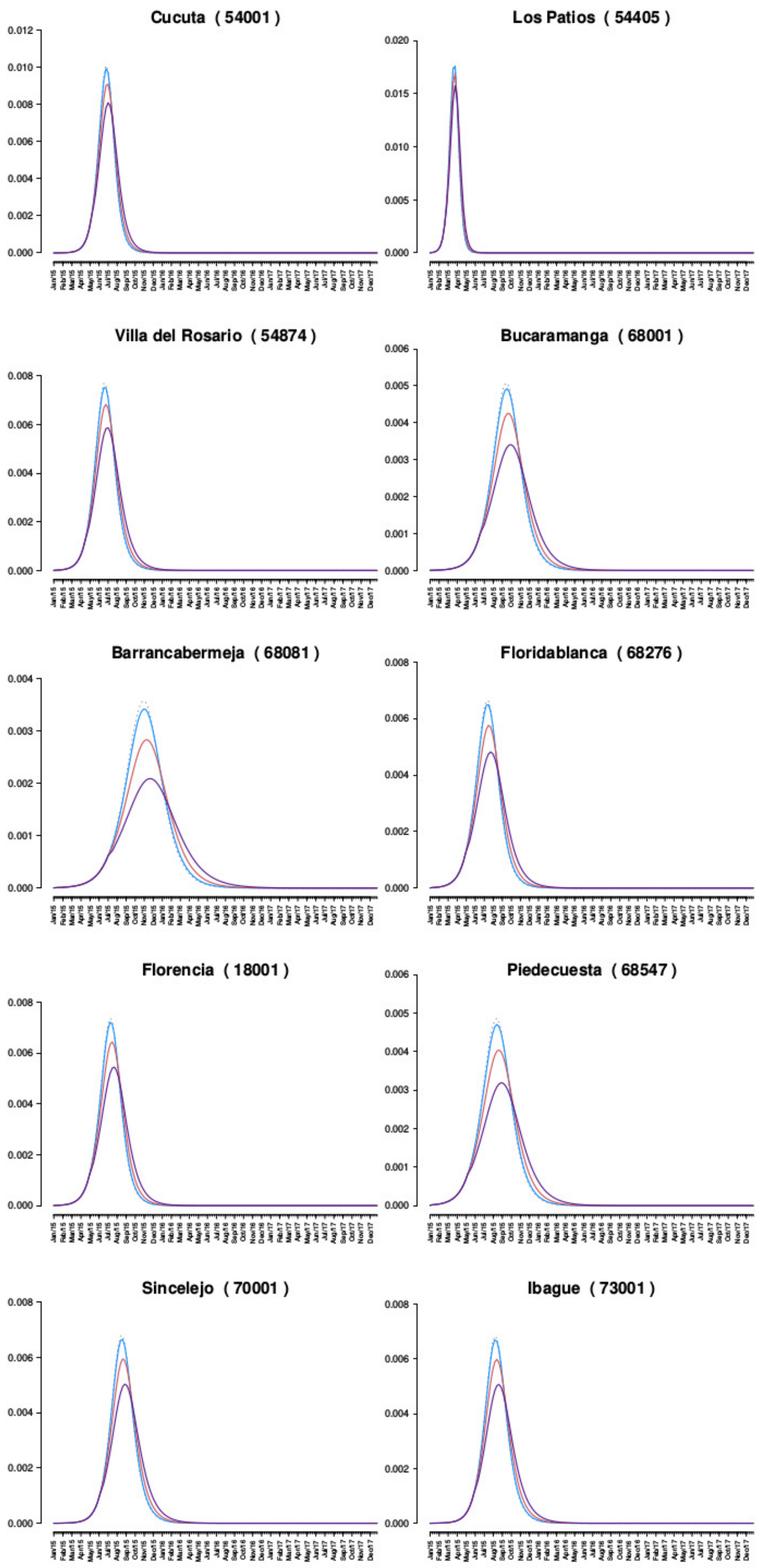

Figure 11. Comparison of the proportion of symptomatic infections of the best fit and the behavior of the epidemics when policies are implemented at th = 20%. Y-axis is a proportion (infected / at risk population), and the x-axis is time (in weeks). Best fit is dashed line, and policy 1,2, and 3 are colored blue, red, and purple, respectively.

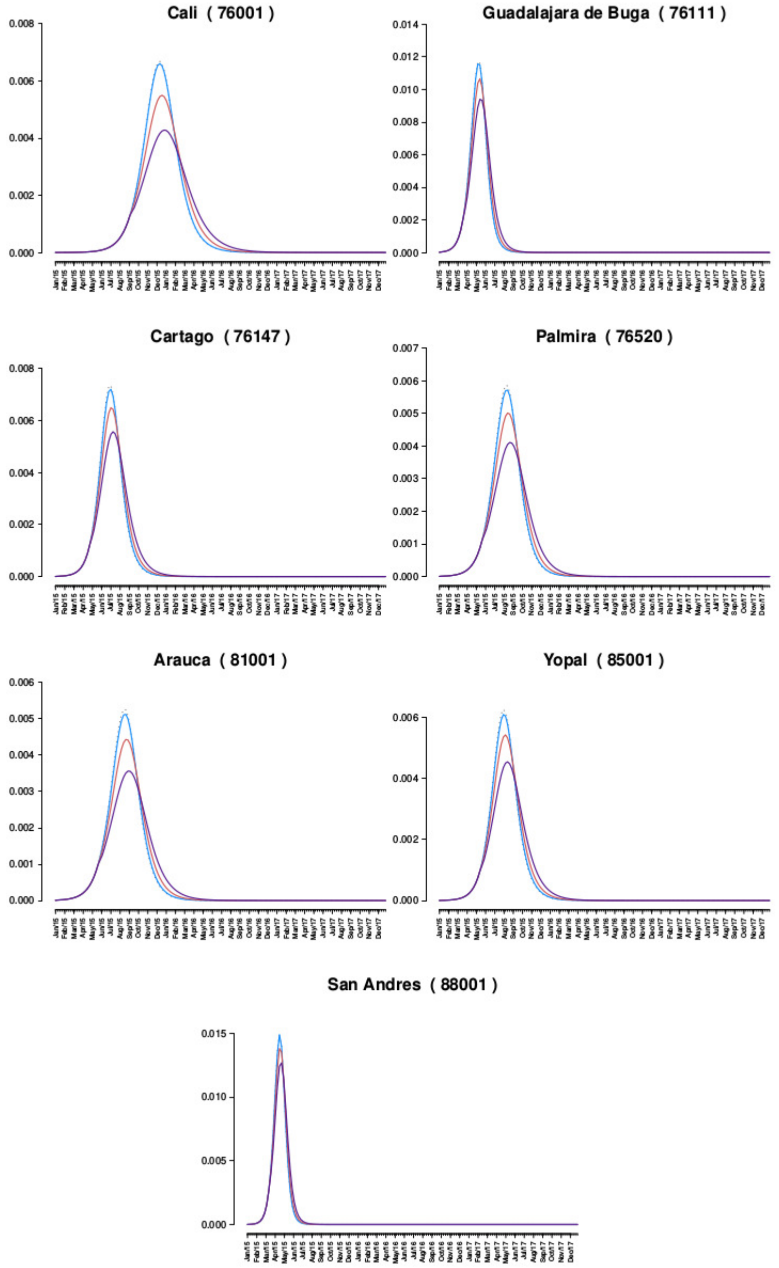

Figure 12. Comparison of the proportion of symptomatic infections of the best fit and the behavior of the epidemics when policies are implemented at th = 20%. Y-axis is a proportion (infected / at risk population), and the x-axis is time (in weeks). Best fit is dashed line, and policy 1,2, and 3 are colored blue, red, and purple, respectively.

7 Impact of Policies $th = 50\%$

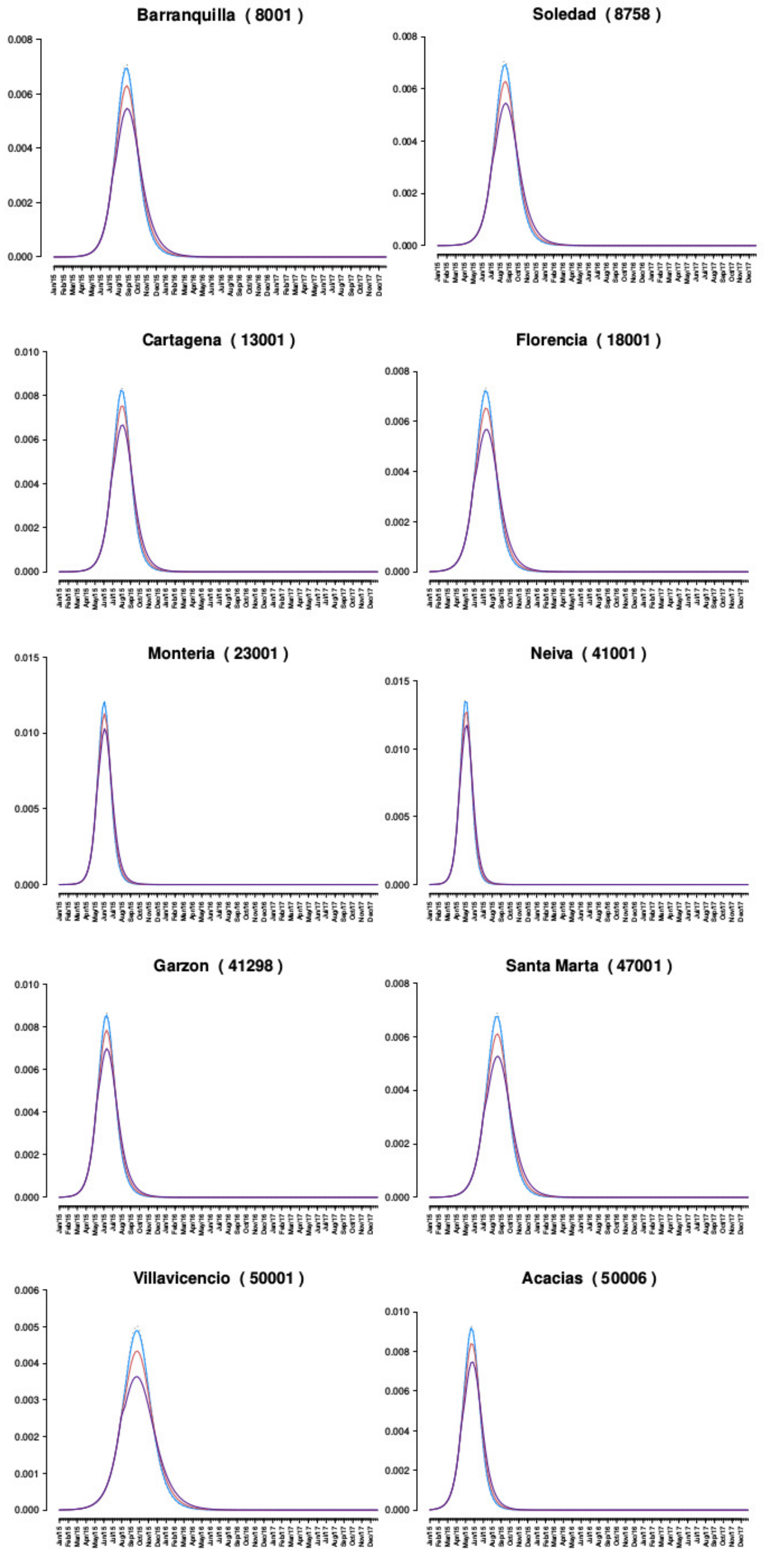

Figure 13. Comparison of the proportion of symptomatic infections of the best fit and the behavior of the epidemics when policies are implemented at th = 50%. Y-axis is a proportion (infected / at risk population), and the x-axis is time (in weeks). Best fit is dashed line, and policy 1,2, and 3 are colored blue, red, and purple, respectively.

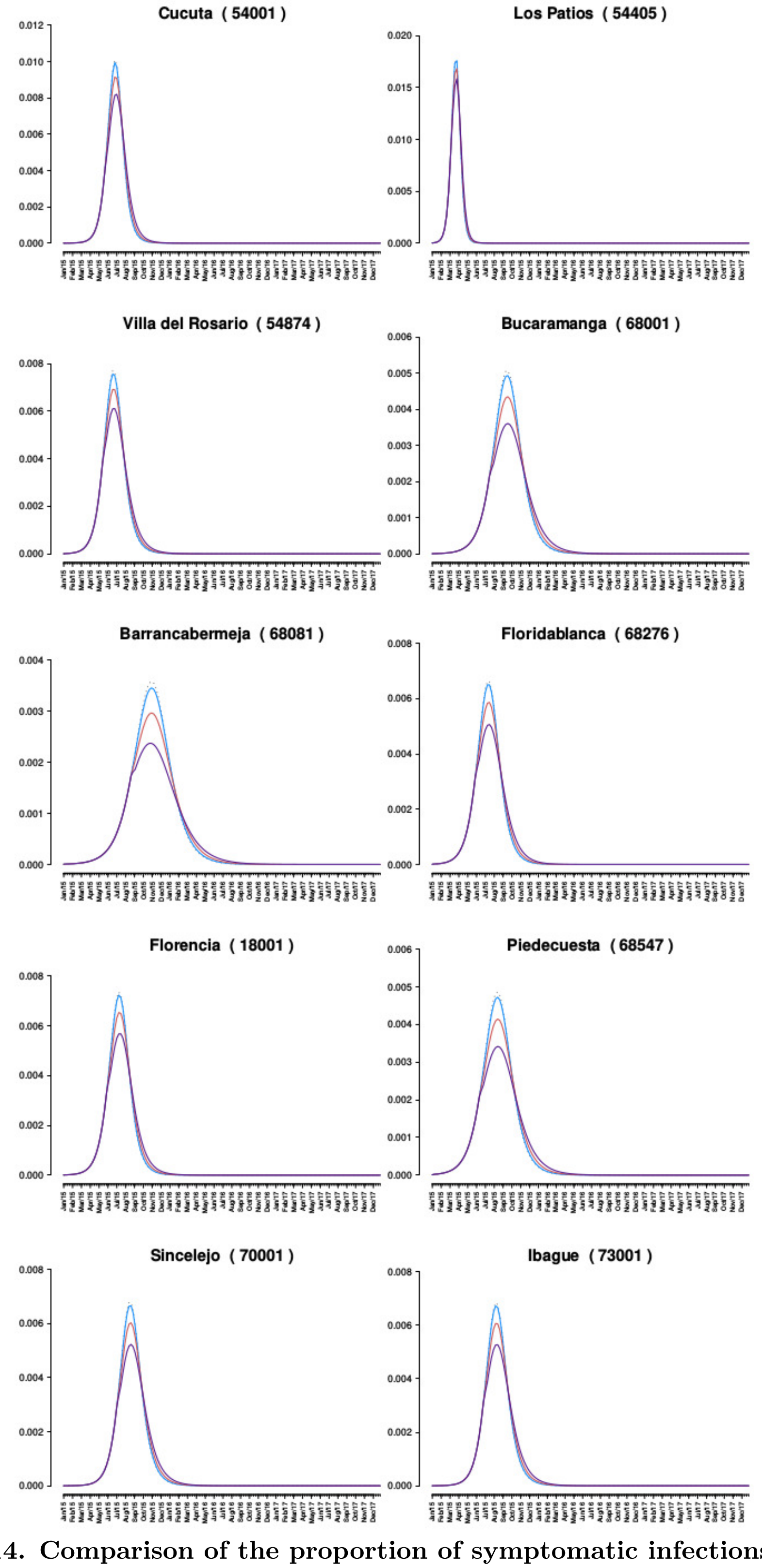

Figure 14. Comparison of the proportion of symptomatic infections of the best fit and the behavior of the epidemics when policies are implemented at th = 50%. Y-axis is a proportion (infected / at risk population), and the x-axis is time (in weeks). Best fit is dashed line, and policy 1,2, and 3 are colored blue, red, and purple, respectively.

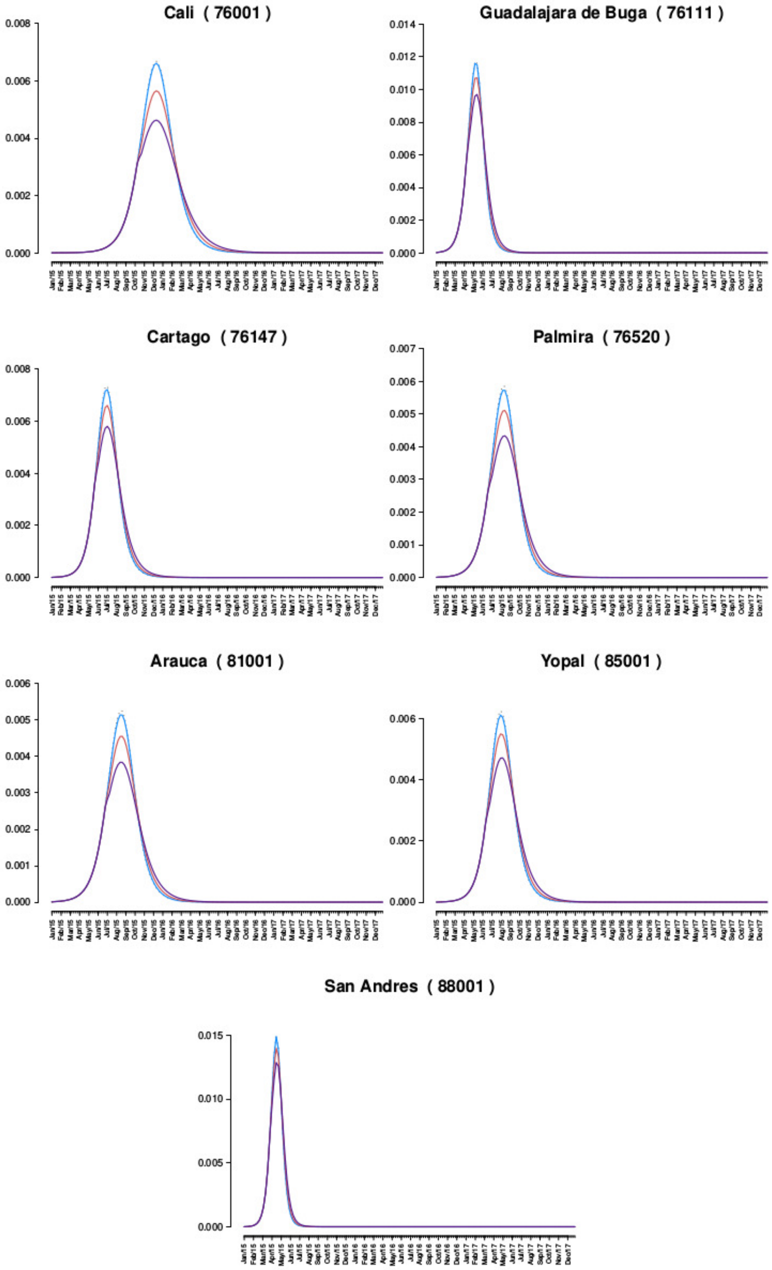

Figure 15. Comparison of the proportion of symptomatic infections of the best fit and the behavior of the epidemics when policies are implemented at th = 50%. Y-axis is a proportion (infected / at risk population), and the x-axis is time (in weeks). Best fit is dashed line, and policy 1,2, and 3 are colored blue, red, and purple, respectively.

8 Impact of Policies $th = 80\%$

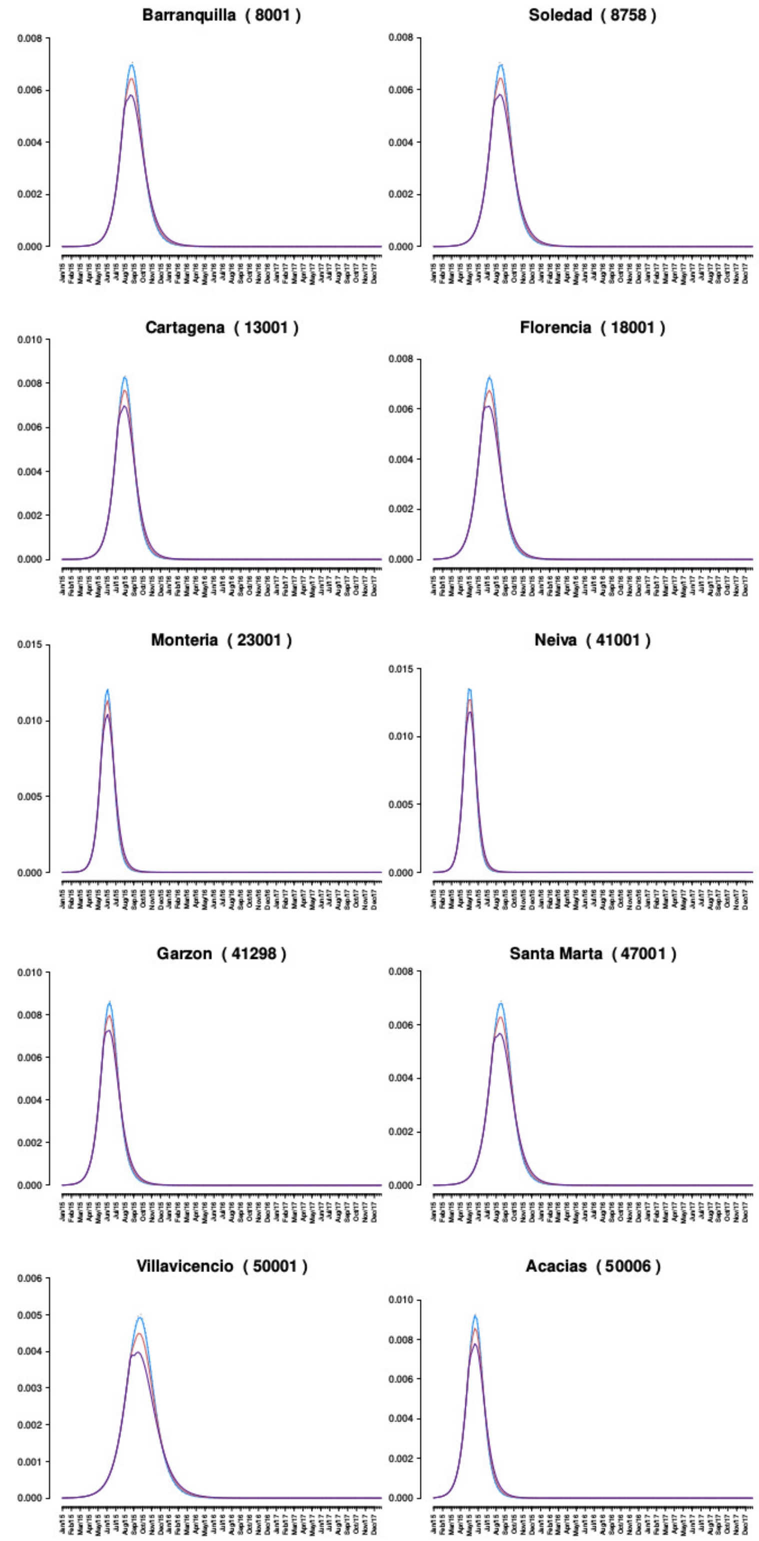

Figure 16. Comparison of the proportion of symptomatic infections of the best fit and the behavior of the epidemics when policies are implemented at th = 80%. Y-axis is a proportion (infected / at risk population), and the x-axis is time (in weeks). Best fit is dashed line, and policy 1,2, and 3 are colored blue, red, and purple, respectively.

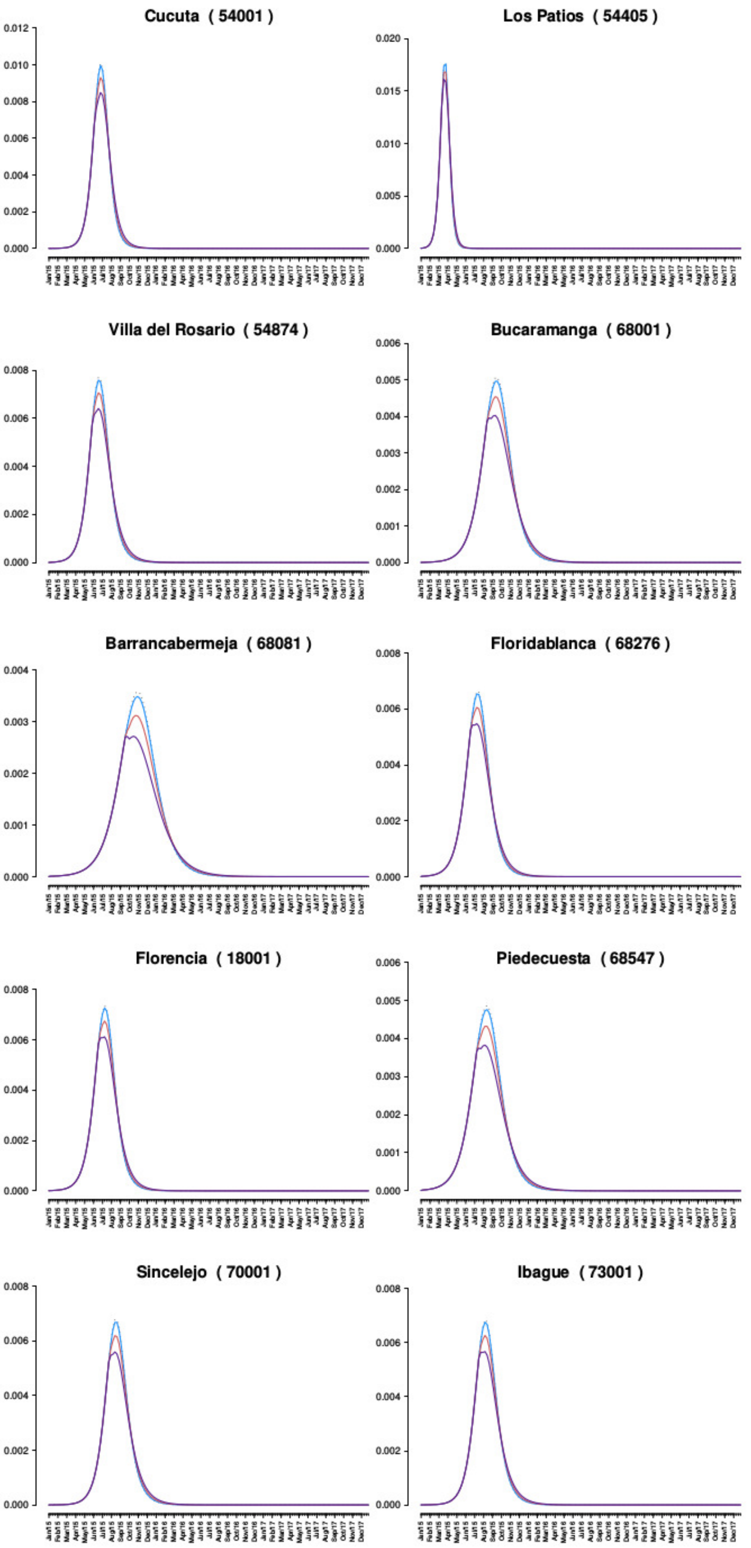

Figure 17. Comparison of the proportion of symptomatic infections of the best fit and the behavior of the epidemics when policies are implemented at th = 80%. Y-axis is a proportion (infected / at risk population), and the x-axis is time (in weeks). Best fit is dashed line, and policy 1,2, and 3 are colored blue, red, and purple, respectively.

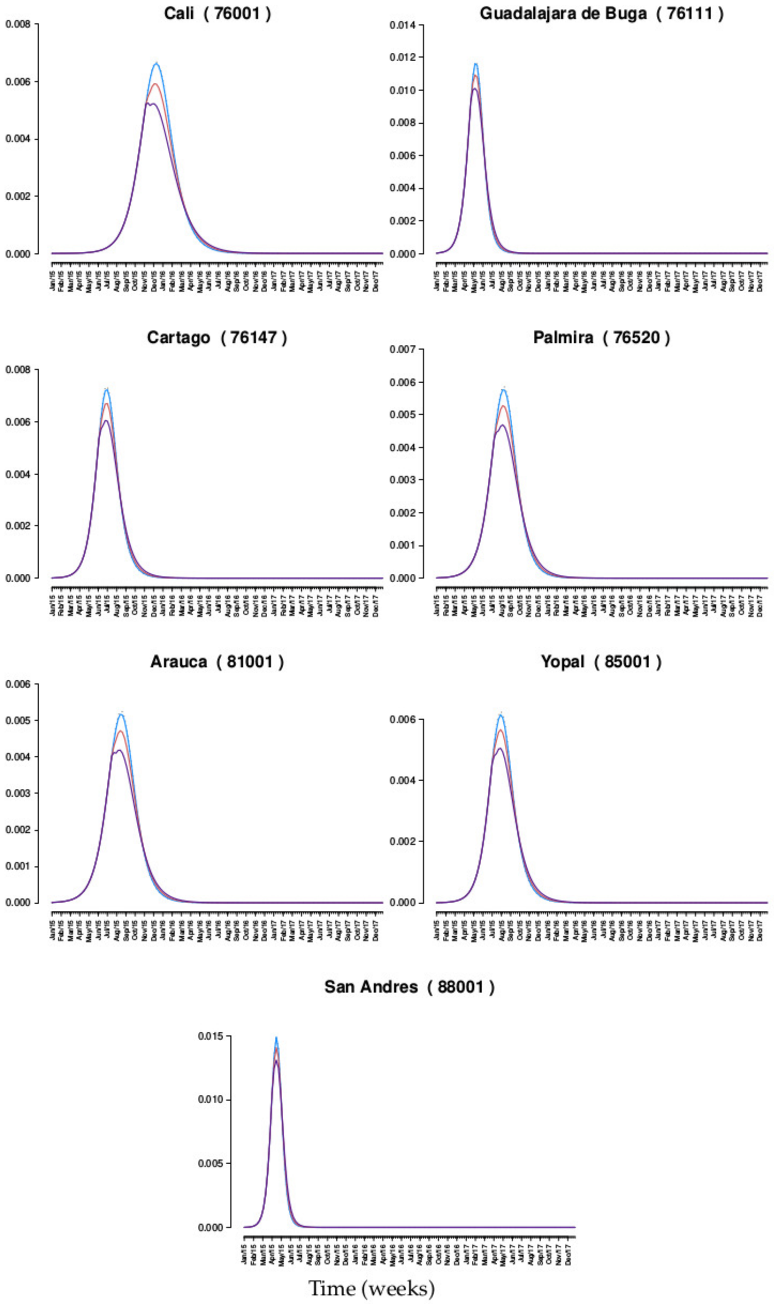

Figure 18. Comparison of the proportion of symptomatic infections of the best fit and the behavior of the epidemics when policies are implemented at th = 80%. Y-axis is a proportion (infected / at risk population), and the x-axis is time (in weeks). Best fit is dashed line, and policy 1,2, and 3 are colored blue, red, and purple, respectively.

9 Varying Risky Sexual Behavior

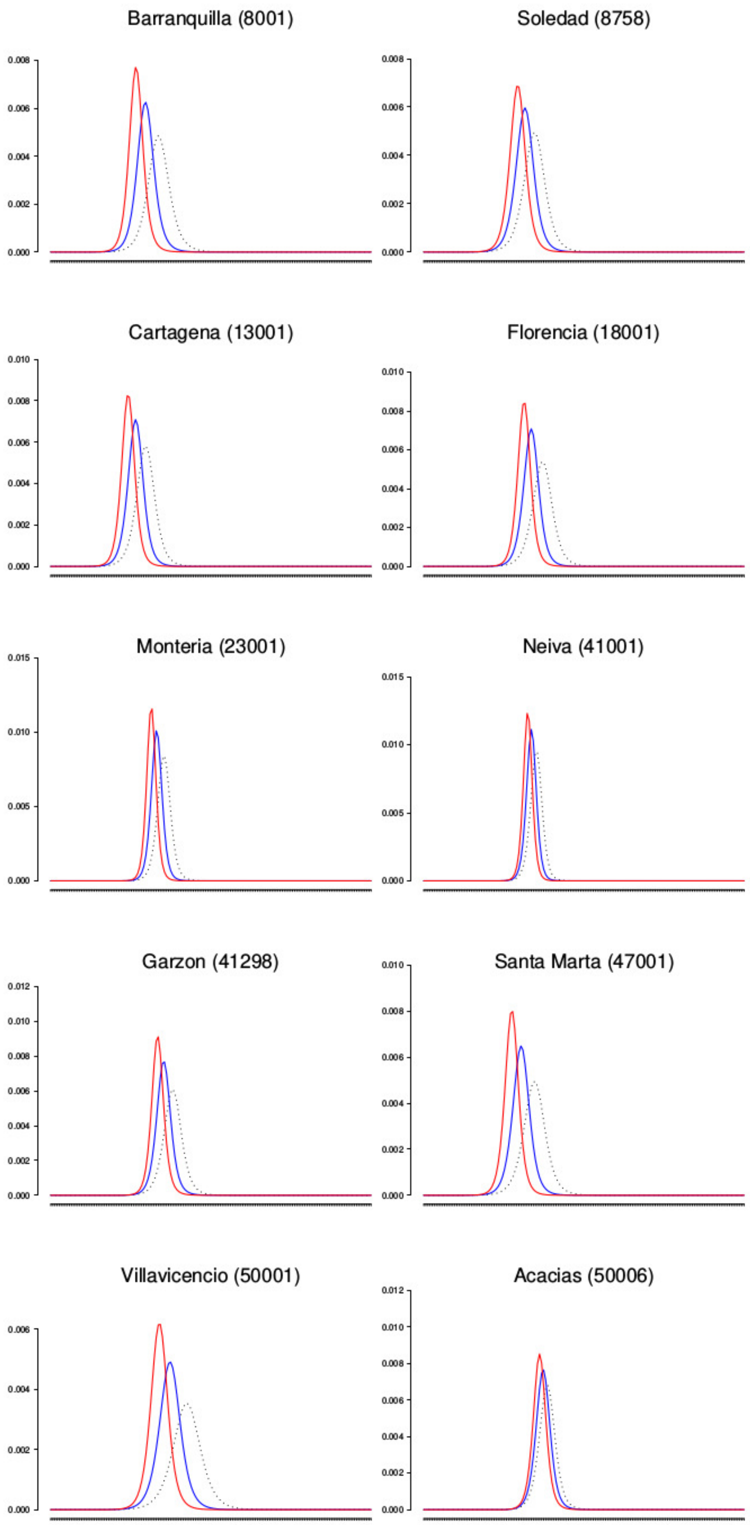

Figure 19. Comparison of the proportion of symptomatic infections of the best fit and the behavior of the epidemics when the number of risky sexual interactions increases. Y-axis is a proportion (infected / at risk population), and the x-axis is time (in weeks). Best fit is dashed line, blue corresponds to = 0.10, and red is = 0.20.

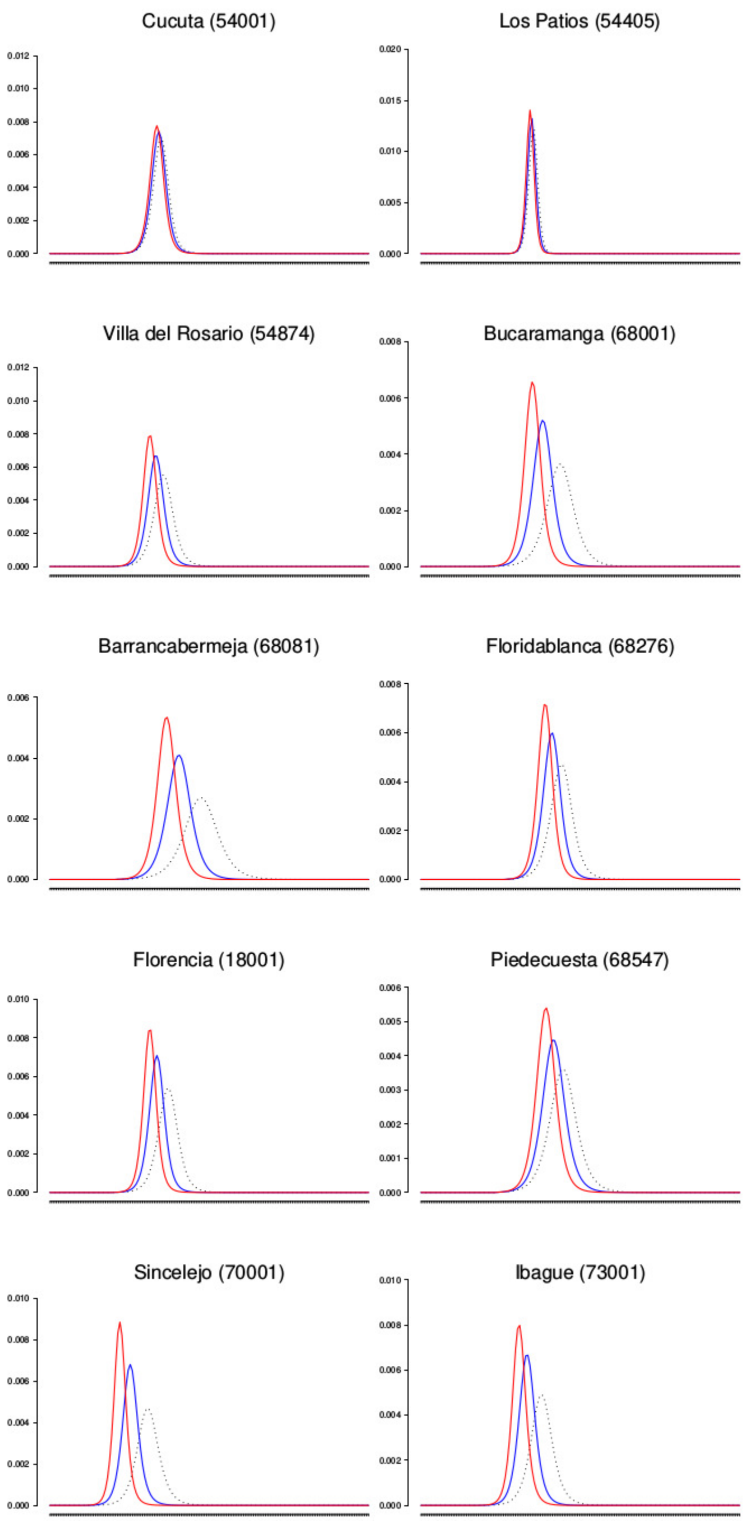

Figure 20. Comparison of the proportion of symptomatic infections of the best fit and the behavior of the epidemics when the number of risky sexual interactions increases. Y-axis is a proportion (infected / at risk population), and the x-axis is time (in weeks). Best fit is dashed line, blue corresponds to $= 0.10$, and red is $= 0.20$.

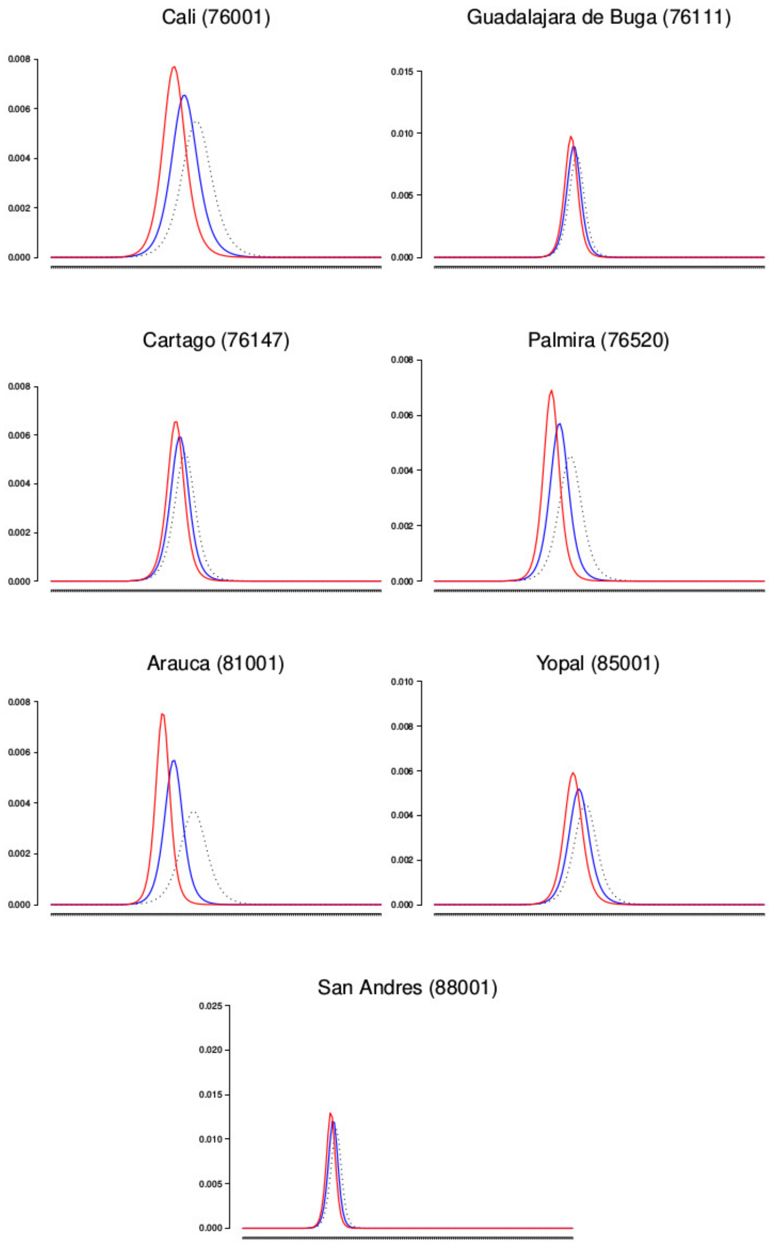

Figure 21. Comparison of the proportion of symptomatic infections of the best fit and the behavior of the epidemics when the number of risky sexual interactions increases. Y-axis is a proportion (infected / at risk population), and the x-axis is time (in weeks). Best fit is dashed line, blue corresponds to $= 0.10$, and red is $= 0.20$.
